# γ-proteobacteria eject their polar flagella under nutrient depletion, retaining flagellar motor relic structures

**DOI:** 10.1371/journal.pbio.3000165

**Published:** 2019-03-19

**Authors:** Josie L. Ferreira, Forson Z. Gao, Florian M. Rossmann, Andrea Nans, Susanne Brenzinger, Rohola Hosseini, Amanda Wilson, Ariane Briegel, Kai M. Thormann, Peter B. Rosenthal, Morgan Beeby

**Affiliations:** 1 Department of Life Sciences, Imperial College London, United Kingdom; 2 Institut für Mikrobiologie und Molekularbiologie, Justus-Liebig-Universität Giessen, Giessen, Germany; 3 Structural Biology of Cells and Viruses Laboratory, The Francis Crick Institute, London, United Kingdom; 4 Institute of Biology, University of Leiden, Leiden, the Netherlands; Indiana University, UNITED STATES

## Abstract

Bacteria switch only intermittently to motile planktonic lifestyles under favorable conditions. Under chronic nutrient deprivation, however, bacteria orchestrate a switch to stationary phase, conserving energy by altering metabolism and stopping motility. About two-thirds of bacteria use flagella to swim, but how bacteria deactivate this large molecular machine remains unclear. Here, we describe the previously unreported ejection of polar motors by γ-proteobacteria. We show that these bacteria eject their flagella at the base of the flagellar hook when nutrients are depleted, leaving a relic of a former flagellar motor in the outer membrane. Subtomogram averages of the full motor and relic reveal that this is an active process, as a plug protein appears in the relic, likely to prevent leakage across their outer membrane; furthermore, we show that ejection is triggered only under nutritional depletion and is independent of the filament as a possible mechanosensor. We show that filament ejection is a widespread phenomenon demonstrated by the appearance of relic structures in diverse γ-proteobacteria including *Plesiomonas shigelloides*, *Vibrio cholerae*, *Vibrio fischeri*, *Shewanella putrefaciens*, and *Pseudomonas aeruginosa*. While the molecular details remain to be determined, our results demonstrate a novel mechanism for bacteria to halt costly motility when nutrients become scarce.

## Abstract 2

When chronically deprived of nutrients, γ-proteobacteria eject their flagella and insert a novel plug into the remains of their flagellar motors, presumably to prevent leakage through the outer membrane.

## Author summary

In the face of starvation, bacteria must minimize their energy use. Here, we describe our unexpected finding that some bacteria take the drastic measure of ejecting their flagella in response to nutrient deficiency. Bacteria continually assemble flagella as propellers—unrelated to eukaryotic flagella—rotated by rotary motors embedded in the cell; continual rotation and assembly can consume up to 3% of a bacterium’s energy. Using electron cryo-tomography, a technique that provides high-resolution 3D images of intact bacteria, we were surprised to find partial flagellar motors in bacterial cells that were rare when nutrients were abundant but became common when nutrients were scarce. A variety of clues led us to hypothesize that these structures were relics of motors whose flagella had been ejected, which we confirmed using a genetic approach. Curiously, flagellar relics—which would otherwise be open portals through which the contents of the bacterial periplasm could leak—were plugged by an unidentified protein, presumably as a preservation measure. We speculate that flagellar ejection saves the bacterium from the costs of continuously assembling and rotating its flagella, as a last-ditch survival attempt. Our work provides a striking example of evolution arriving at a functional yet unintuitive solution to a problem.

## Introduction

Many bacteria switch to a nonmotile lifestyle in stationary phase to conserve energy [1], but how this switch is accomplished is poorly understood. The most widespread motility device used by bacteria is the flagellum, found in approximately two-thirds of bacteria [2]. Bacterial flagellar filaments are helical propellers that extend several microns from the cell from a periplasm-spanning rotary motor; rotation of the filament by a transmembrane rotary motor exerts thrust that propels the bacterium forward. Motor torque is generated by harnessing ion flux across the inner membrane; this torque is first transmitted to a periplasm-spanning rod, then to a flexible extracellular hook, and finally to the filament. The rod exits the outer membrane through dedicated P- and L-rings that act as channels through the peptidoglycan layer and outer membrane, respectively. Although the flagellum is vital for migration to favorable environments, sites of biofilm formation, or sites of infection, it is counterproductive for the cell to retain a functional flagellum during nutrient depletion, and stopping motility is preferable to resource exhaustion.

Methods for deactivation of flagella in different environments other than nutrient depletion are diverse across those bacteria studied to date. *Rhodobacter sphaeroides* has a unidirectional flagellum that is stopped by a “molecular brake” for navigation [3], while *Bacillus subtilis* uses a “molecular clutch” to stop flagellum rotation and swimming for biofilm formation [4]. The *Salmonella enterica* serovar Typhimurium (“*Salmonella*”) and *Escherichia coli* motors are proposed to be inactivated by a “backstop brake”, YcgR, a cyclic di-GMP (c-di-GMP) binding protein [5,6], while *P*. *aeruginosa* modulates its motility via a YcgR homologue, FlgZ [7]. The α-proteobacterium *Caulobacter crescentus*, meanwhile, actively ejects its single, polar flagellum upon surface sensing [6,7] in order to build an adhesive stalk for surface adhesion.

Little is currently known about how the widespread polar-flagellated γ-proteobacteria modulate motility. This group includes a diverse set of pathogens occupying both sessile and planktonic niches, including human pathogens *V*. *cholerae* and *P*. *shigelloides*, opportunistic pathogens *P*. *aeruginosa* and *S*. *putrefaciens*, and nonpathogenic members including the squid symbiont *V*. *fischeri*. These γ-proteobacteria have one or more polar flagella whose motors are powered by Na^+^-ions; these motors are both faster [8] and provide higher torque [8,9] than the model peritrichous (positioned over the entire cell surface) flagellar motors from *Salmonella* and *E*. *coli*; polar Na^+^-driven motors assemble at only one pole until division. Na^+^-driven polar motors have a number of structural differences to the well-studied enteric-like motors [9–11]. Most striking in subtomogram averages is the addition of periplasmic (H-ring and T-ring) and outer membrane (basal) disks [2,9]. The T-ring, made up of MotX and MotY, contributes to the assembly and scaffolding of the stator complexes (PomA/PomB). Many γ-proteobacteria also incorporate a sheath, an outer membrane extension that encapsulates the flagellum. The H-ring and basal disk, composed of FlgO, FlgP, and FlgT, have recently been shown to assist in outer membrane penetration in these bacteria [12], although their function is likely broader than sheath formation, given that many unsheathed bacteria also retain an H-ring.

Here, we describe work showing that γ-proteobacteria eject their polar Na^+^-driven flagella in response to nutrient depletion. The ejected flagella leave a “relic” of the ejected motor in the outer membrane composed of the P-, L-, H-, and T-rings and the basal disk. During this transition, a previously undescribed plug is incorporated into the relic. This is likely an active mechanism to block periplasmic leakage. We speculate on the nature of the novel flagellar plug and the significance of flagellar ejection. Note that this work was previously available as a preprint [13]; a similar preprint concluded that “relics” are instead outer-membrane assembly precursors [14].

## Results

### γ-proteobacterial motility slows as cell density increases

**Fig 1:**
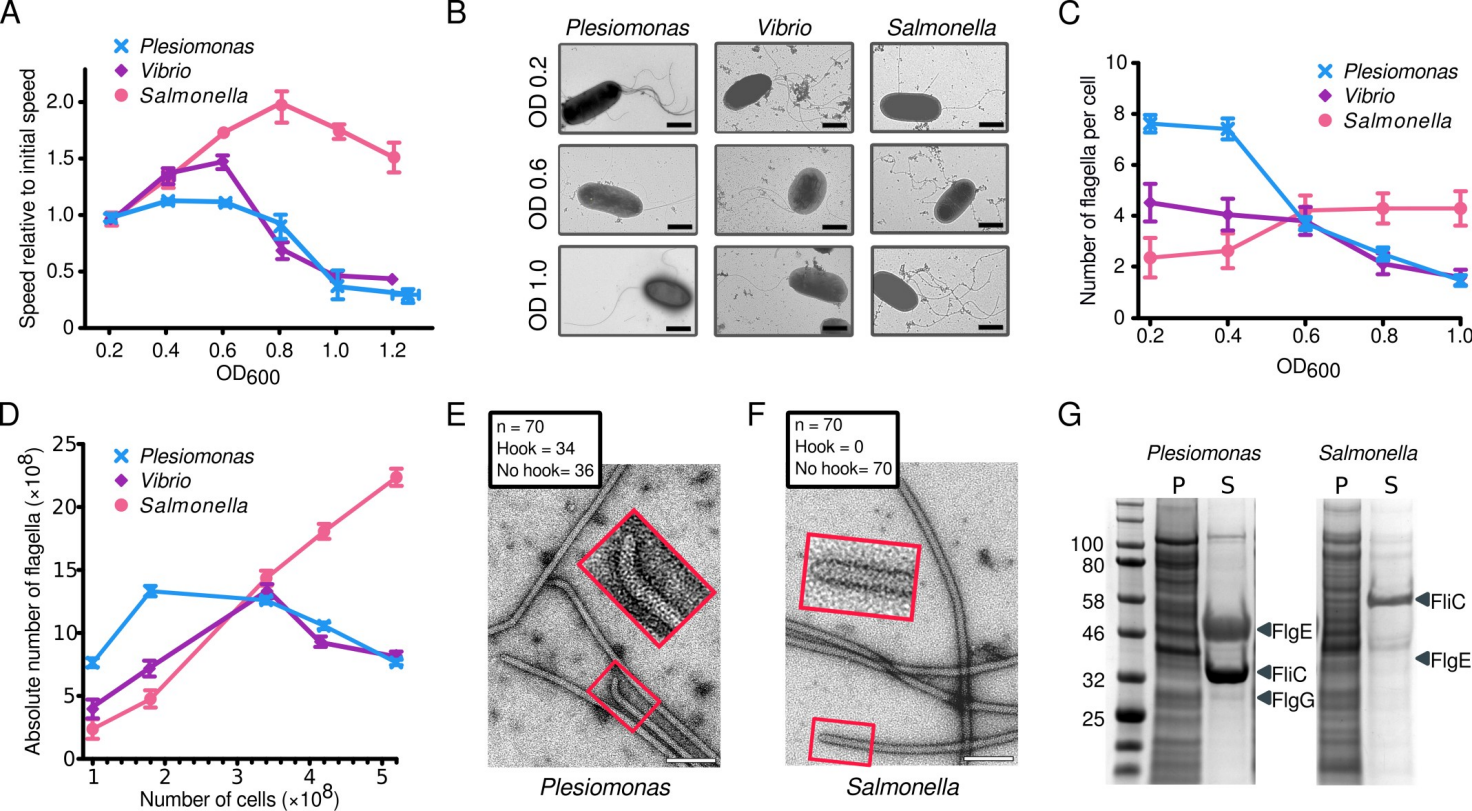
γ-proteobacteria swimming slows at later growth stages due to loss of flagella. (**A**) Swimming speeds of *P*. *shigelloides*, *V*. *fischeri*, and *S*. *enterica* sv. Typhimurium at increasing cell density. Speed relative to initial speed at OD_600_ 0.2 are represented. Error bars indicate standard error. (B) Representative negative-stain EM images of cells grown to three different cell densities of *P*. *shigelloides* and *V*. *fischeri*, revealing loss of polar Na^+^-driven flagella yet no loss of peritrichous *Salmonella* flagella. Scale bars are 1 μm. (C) Mean number of flagella, counted from 150 cells (50 per biological replicate) at increasing cell densities suggests loss of polar flagella. The error bars indicate a 95% t-based confidence interval. (D) The absolute number of attached flagella in the population calculated from the mean number of flagella and CFU suggests flagellar ejection by *P*. *shigelloides* and *V*. *fischeri*. The error bars indicate a 95% t-based confidence interval. (E) Negative-stain EM images of flagella recovered from the supernatant of stationary phase *P*. *shigelloides* confirms consistent flagellar loss from the base of the hook. Inset shows close-up of a hook. Scale bar is 100 nm. (F) Negative-stain EM images of flagella recovered from the supernatant of stationary phase *Salmonella*. Inset shows close-up of a broken filament; no hooks were observed in isolated *Salmonella* flagella. Scale bar is 100 nm. (G) SDS-PAGE gel of *P*. *shigelloides* and *Salmonella* cells grown to stationary phase confirms ejection of polar Na^+^-driven flagella at the base of their hook. Polar FliC (filament) and FlgE (hook) proteins are seen in the S of the *P*. *shigelloides* culture. Underlying data in S1 Data. CFU, colony-forming unit; EM, electron microscopy; OD, optical density; P, pellet; S, supernatant.

While tracking the swimming speeds of the γ-proteobacterium *P*. *shigelloides*, we noticed that bacteria with multiple Na^+^-driven polar flagella showed a significant decrease in swimming speed when grown to later growth stages (Fig 1A). Video tracking of individual bacterial cells revealed that *P*. *shigelloides* swam at 40 μm s^−1^ between optical density (OD) 0.2 and OD approximately 0.7 before swimming speeds sharply dropped at OD 0.8, down to 12 μm s^−1^ at OD 1.0. Furthermore, the percentage of active swimmers dropped from over 95% at early growth stage up to OD 0.6 to approximately 5% by OD 1.0. Another γ-proteobacterium, *V*. *fischeri*, displayed the same characteristic behavior (Fig 1A). Strikingly, however, the model enteric bacterium *Salmonella* that uses a different family of flagellar motors continued swimming as well as, if not faster than, *Salmonella* cells at OD 0.2 when cultured to higher cell densities (Fig 1A).

### Decreased swimming speed is due to loss of flagella

In the course of a previous study [9], we noted that flagellation levels in γ-proteobacteria are highest early in their growth stage (low cell density). To determine whether the decrease in swimming speed of γ-proteobacteria was due to differences in flagellation levels at different growth stages, flagella were counted in negative-stain electron microscopy (EM) images of cells to assess flagellation levels (Fig 1B). Time courses of both *P*. *shigelloides* and *V*. *fischeri* cells grown for longer periods of time (at increasing cell densities) showed decreasing numbers of flagella per cell (Fig 1B and 1C), and cells grown overnight into late stationary phase had no flagella. *Salmonella*, in contrast, had increasing or stable numbers of flagella at later growth stages (higher cell densities).

We sought to distinguish whether solely flagellar synthesis is down-regulated or whether flagella are also actively disassembled in γ-proteobacteria at high cell densities. Plotting the absolute number of flagella in the entire population indicated that flagella are indeed lost. The number of cells in a population was determined by calculating colony-forming units (CFUs) and relating this to the mean number of flagella per cell as counted by EM (Fig 1D). The absolute number of flagella in the *P*. *shigelloides* and *V*. *fischeri* populations declined over time, demonstrating that bacteria are losing flagella faster than they are synthesizing them (Fig 1D). The *Salmonella* positive control, in contrast, showed an increase in the absolute number of flagella in the population in direct correlation with CFU count, as assembly continued and no ejection occurred in later growth stages (at higher cell densities).

### Used γ-proteobacterial growth medium contains free flagella with hooks at one end

To test our speculation that flagella are ejected, we determined whether supernatant of *P*. *shigelloides* cells grown to stationary phase contains released flagella. Polyethylene glycol (PEG) precipitation revealed that *P*. *shigelloides* growth medium indeed contained free flagella (Fig 1E). Curiously, 50% of all flagellar ends had an attached hook. Given that a flagellum only has a hook attached at one of its ends, we conclude that, effectively, all isolated *P*. *shigelloides* flagella have attached hooks, similar to ejected flagella from *Caulobacter crescentus* [15]. Flagellar filaments were also recovered from the supernatant of a *Salmonella* control (Fig 1F). Strikingly, however, no hooks were observed on any of the 70 randomly imaged *Salmonella* filament ends. This suggests random instead of determinate sites of filament breakage in *Salmonella*. As the number of flagella on *Salmonella* cells increases or remains stable at high density (Fig 1C), the broken flagellar filaments must regrow, which is in agreement with previous studies [16,17].

### Shed hook–filament structures accumulate in *P*. *shigelloides* supernatant

To verify that *Salmonella* filaments are sheared midfilament, whereas *P*. *shigelloides* filaments are cleaved at the base of the hook, flagella were recovered from cell cultures and analyzed by SDS-PAGE. Cells grown to high OD were removed by pelleting, and flagella were recovered from the remaining supernatant by PEG precipitation before SDS-PAGE analysis. Similar samples were collected from *Salmonella* cultures and volumes adjusted to match *P*. *shigelloides* cell count. The *Plesiomonas* supernatant had significant bands at molecular weights corresponding to both the polar flagellin (FliC) and hook (FlgE), as well as a faint band at the expected size of the distal rod (FlgG). The *Salmonella* supernatant contained a weaker flagellin band but lacked bands at the molecular weights corresponding to the *Salmonella* hook (FlgE) or rod (Fig 1G). This confirms that *Plesiomonas* cells consistently lose their flagella at the base of the hook, while *Salmonella* flagella accumulation in the supernatant is due to shearing midfilament. We conclude that this consistent behavior in response to a specific environment, i.e., late growth stage and high cell densities, is best explained by an ejection mechanism.

### Partial relics of flagella remain at the cell pole at high OD

**Fig 2:**
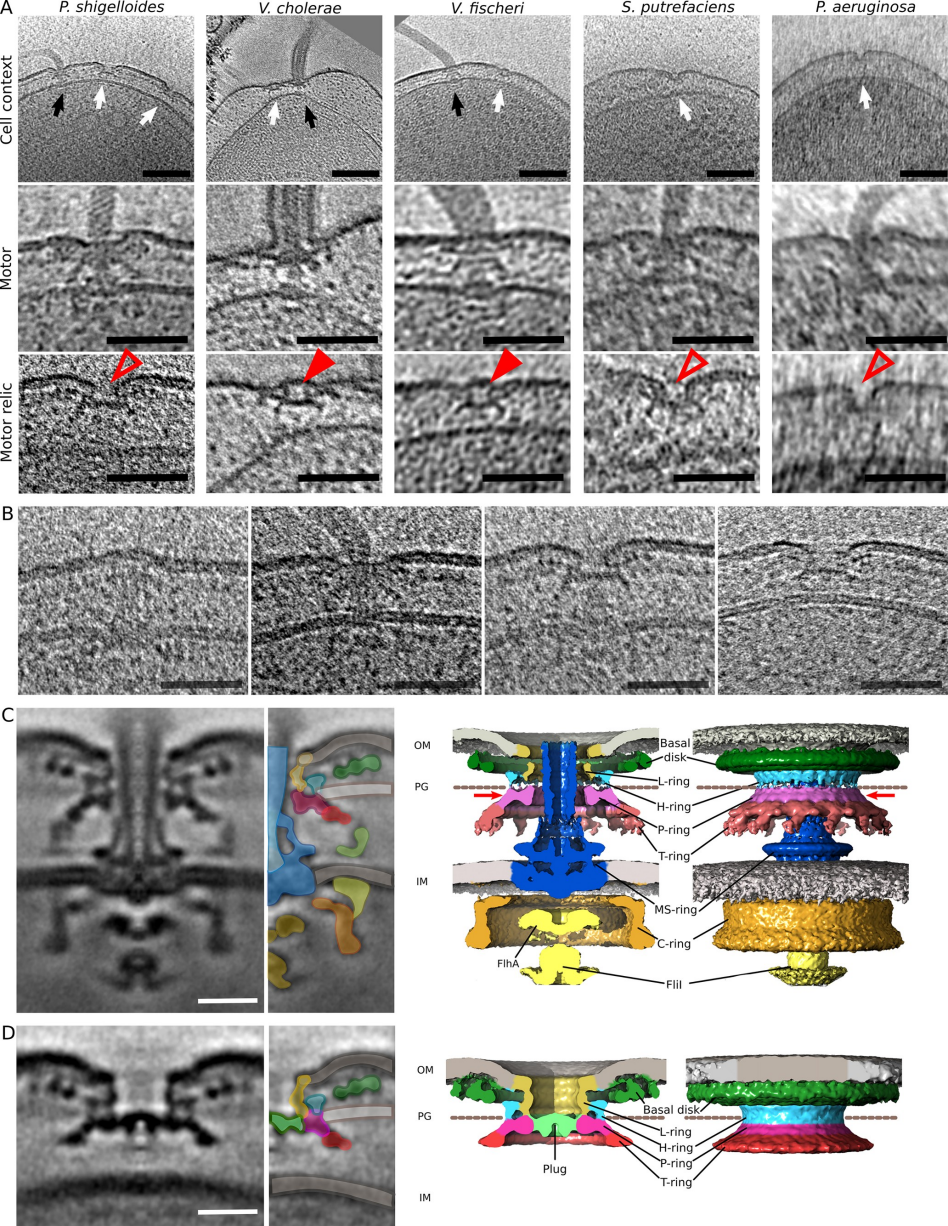
Old γ-proteobacterial cell poles bear the relics of ejected flagella. (A) Top row: slices through tomograms showing flagella and relic structures in five γ-proteobacteria. (Left to right): *P*. *shigelloides*, *V*. *cholera*, *V*. *fischeri*, *S*. *putrefaciens*, and *P*. *aeruginosa*. Top row: black arrows point to full flagella, and white arrows point to relics. Scale bar is 100 nm. Middle row: example subtomograms of full flagella in the five species. Scale bar is 50 nm. Bottom row: example subtomograms of relics in the five species. Scale bar is 50 nm; empty red arrowheads highlight open relic structures from unsheathed bacteria, filled red arrowheads highlight membrane-capped relic structures from sheathed bacteria. (B) Assembly and disassembly states are seen in *P*. *shigelloides* tomograms, including precursors that differ from relic structures. Scale bar is 50 nm. (C) Subtomogram average of the *P*. *shigelloides* polar flagellar motor. (Left to right): central slice through the subtomogram average, interpretive colored overlay relating subtomogram average to isosurface representation, cutaway isosurface rendering of subtomogram average, and whole isosurface rendering. Dotted line represents likely peptidoglycan location; red arrow highlights where the plug density sits in the relic. Scale bar is 15 nm. The structure is deposited to the Electron Microscopy Data Bank as EMDB-4570. (D) Corresponding subtomogram average (left) with isosurface (right) of the *P*. *shigelloides* polar relic structure. Scale bar is 15 nm. The structure is deposited to the Electron Microscopy Data Bank as EMDB-4569.

The hypothesis that γ-proteobacteria eject their flagella led us to speculate that a partial flagellar motor structure would remain after ejection of the filament and hook. Cryo-electron tomograms were collected of *P*. *shigelloides* cells grown to either early or late growth stages (low or high cell density). Consistent with our hypothesis, partial flagellar structures were seen to remain at the pole of cells grown to high OD, with fewer in cells grown to low OD (Fig 2A, left column). In all cases, partial flagellar structures were only observed at the flagellated cell pole. Our swimming speed and flagellar ejection results led us to speculate that these structures are the “relics” of once fully-functioning flagella that were subsequently ejected in late-growth stage (high OD) cultures, leaving only the outer-membrane rings (the P-, L-, H-, and T-rings and the basal disk) assembled. The P- and L-rings, which are integral parts of the relic structure, require a rod and chaperone for assembly; the P- and L-rings are incapable of assembling in the absence of an assembled rod [18], further consistent with these structures being relics of previously full flagella.

The two data sets demonstrated that at high OD, relics are enriched while flagella are depleted, correlating with our observations of flagellar ejection (Table 1). In cells imaged at OD 0.25, flagella were predominant: 43% of cell poles had exclusively intact flagella, 48% had both intact flagella and relics, and 9% had no structures; the average number of intact flagella per pole was 5.5; if the cells had relics, the average number of relics was 2. In cells imaged at OD 1.0, relics were predominant: none of the cells had intact flagella alone; 36% had both relics and intact flagella, 12% had exclusively relics, and 52% had neither relics nor flagella; and in contrast to cells grown to low OD, if the cells had intact flagella, the average seen was 1.3, while the average number of relics was 3.3. Note that because relics are harder to detect in the cryo-tomograms since they are relatively small and lack a flagellum, we may not have identified all relics that were present in the imaged cells. Consistent with this, imaging lysed cells grown to OD 1 revealed substantially more relics than we could detect in intact cells (up to 20 at a pole) (S1 Fig). This data set was not used in Table 1, however, as we cannot deconvolute any effect of cell lysis on relic appearance. Nevertheless, this result confirms that many relics accumulate at the cell pole. The results in Table 1 link OD to presence of the relic structures and demonstrate that not only are flagella being ejected, but assembly is also halted, as many cells harvested at high OD have neither flagella nor relics, indicating newly formed poles that have not yet assembled a single flagellum.

**Table 1:** Appearance of outer membrane relic structures is related to cell density. Number of filaments and outer membrane relics counted from tomograms of *P*. *shigelloides* cells grown to OD_600_ 0.25 (*N* = 44) or OD_600_ 1.0 (*N* = 25).

OD_600_	Number of poles (*N*)	Poles with filaments and relics (%)	Poles with only filaments (%)	Poles with only relics (%)	Poles with nothing (%)	Average number of filaments from all poles	Average number of relics from all poles	Average number of filaments for poles with filaments	Average number of relics for poles with relics
0.25	44	21 (47.7)	19 (43.2)	0 (0)	4 (9.1)	5	0.9	5.5	1.9
1	25	9 (36)	0 (0)	3 (12)	13 (52)	0.5	1.6	1.3	3.3

**Abbreviation:** OD, optical density.

### Diverse γ-proteobacteria retain relics of ejected flagella

In order to determine whether this pattern is widespread amongst the γ-proteobacteria beyond *P*. *shigelloides*, we collected cryo-tomograms from four additional γ-proteobacterial species: *V*. *cholerae*, *V*. *fischeri*, *S*. *putrefaciens*, and *P*. *aeruginosa* (Fig 2A, right four columns). Markedly, in all species, we observed similar relic structures in the outer membrane of the cell poles, alongside intact flagella (Fig 2A). This was particularly unexpected in *V*. *cholerae*, *S*. *putrefaciens*, and *P*. *aeruginosa*, given that they only ever assemble one flagellar motor at the pole. Indeed, in one tomogram, we observed four relics alongside a single intact flagellum in *V*. *cholerae*.

Relics from sheathed and unsheathed γ-proteobacteria had different outer-membrane morphologies. Relics from tomograms of sheathed *V*. *cholerae* and *V*. *fischeri* were capped by a continuous sealed outer membrane. Ejection of the flagellum in these bacteria must include shearing of the sheath from the outer membrane and subsequent closure of the torn membrane. Evidently, the unsheathed species *P*. *shigelloides*, *S*. *putrefaciens*, and *P*. *aeruginosa* maintain a portal through the outer membrane at all times with their L-ring that is unperturbed upon flagellar ejection. This suggests an as-yet unidentified difference between the L-rings from sheathed and unsheathed bacteria.

### Multiple assembly intermediate states, distinct from relics, are observed in cryo-tomograms

Alongside relics and intact flagella, multiple intermediate states were captured in our cryo-tomograms (Fig 2B). Early precursor structures include fully formed C-rings and rods but lack P- and L-ring–based outer-membrane disks and external structures. However, by far the most common structures observed were fully formed flagella and relic structures.

### A novel protein plug in flagellar relics suggests a method for preventing periplasmic leakage

To better understand the relationship of relics to intact motors, we determined the structures of both by subtomogram averaging of the *P*. *shigelloides* structures (Fig 2C and 2D). Clear 13-fold symmetry was observed in the stator complexes and MotXY ring of the full motor (S2 Fig), consistent with previous studies [9,11]. The relic structure resembled the outer-membrane flagellar structure, although it lacked bound stator complexes, suggesting that the relic is composed of the same proteins as the outer membrane–associated structures from the flagellar motor. In both structures, the distance between the MotXY ring (T-ring) and the outer membrane was 19 nm, and the diameter of the MotXY ring was 44 nm. In the motor, the diameter of the rod exit hole in the outer membrane was 15 nm, which is the same as in the relic outer membrane that is held open by the L-ring, despite lack of the rod. To confirm that relic structures really are remnants of old flagellar motors, we sought to confirm that they are composed of the same proteins as the outer-membrane portion of the flagellar motor. We purified a His_6_- and mCherry tagged version of *S*. *putrefaciens* MotX, the outermost protein from the T-ring, from membranes by affinity purification. Negative-stain EM and 2D classification of our affinity-purified, MotX-containing particles revealed homogenous top-views. These were comprised of a series of concentric rings, filled in the middle, consistent with top-views of relic subtomogram averages (S3 Fig). The sizes of the rings matched the basal disk (50 nm), T-ring (37 nm), and MotY-ring (29 nm) of *S*. *putrefaciens*. Based on these observations, we conclude that relics are composed of flagellar outer-membrane proteins yet lack flagellar inner membrane, rod, and hook components.

Notably, an extra density in the relic structure plugged the P-ring aperture in the position that was previously occupied by the trans-periplasmic rod. Comparison of the flagellar and relic subtomogram averages confirmed that this density was unambiguously an additional protein absent from intact flagellar motors: while the rod was absent from the relics, the additional relic density observed did not correspond to loss of density elsewhere in the relic, which would result from a conformational change in a previously assembled component protein. Furthermore, this plug appeared to be an obligate part of the relic, as no relics were observed without a plug upon detachment of the axial structure—made of part of the rod, the hook, and filament—from the relic structure. The relics from both sheathed and unsheathed bacteria contained the plug protein regardless of the sealing of the outer membrane in the sheathed flagella. Bioinformatic predictions of potential novel flagellar components based on operon co-occurrence and phylogenetic profiling techniques [19], however, failed to identify suitable candidates, and we were unable to refine relic purification sufficiently for mass spectrometry analysis. We conclude that the plug is an as-yet unidentified protein that plugs the P-ring aperture concomitantly with flagellar ejection to prevent periplasmic leakage.

### Verification that the outer-membrane structure is a flagellar relic and not an assembly precursor

**Fig 3:**
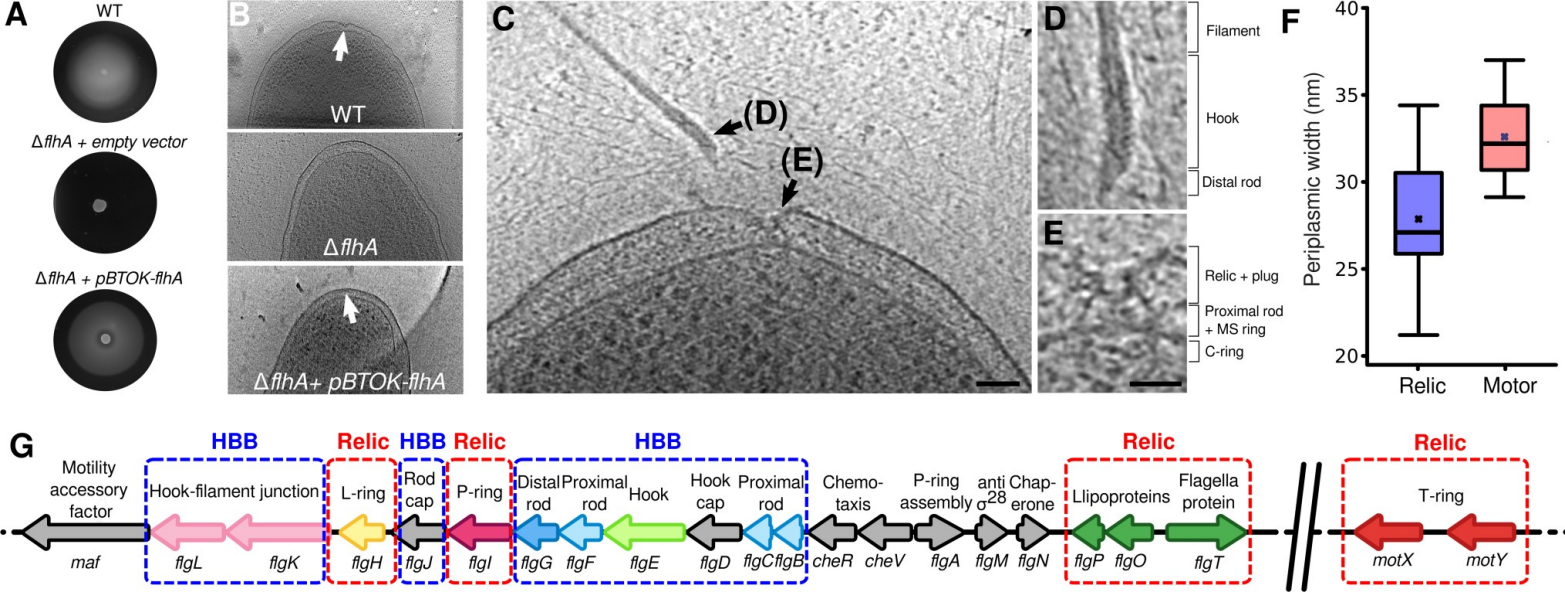
Flagellar partial structures are relics, not assembly intermediates. (A) Soft-agar plates confirm loss of motility upon deletion of *flhA*, regained upon complementation. (B) Representative tomograms of (top to bottom) wild-type *S*. *putrefaciens* with partial structures, Δ*flhA* mutant without partial structures, and return of partial structures upon complementation with pBTOK-*flhA*. (C) Ejection caught in the process. A slice through a tomogram of *S*. *putrefaciens* immediately after an ejection event. The break point of ejection appears to be between the proximal and distal rod. Scale bar is 50 nm. (D) A close-up view of the recently ejected filament, containing the filament, hook, and distal rod. Scale as in next panel. (E) A close-up view of the components remaining in the cell immediately after ejection of the filament, hook, and distal rod. The plug density is already in place, and the proximal rod remains, along with the MS-ring and cytoplasmic components. Scale bar is 25 nm. (F) Periplasmic distance at position of relics or motors. Box is first and third quartile, the line in the box is the median, and the cross is the mean. (G) Rod, hook, and relic genes are interspersed together in an operon. Colors as in Fig 2C and 2E. Underlying data in S1 Data. HBB, hook-basal body.

We sought to verify whether our so-called relic partial flagellar structures were indeed flagellar relics and not flagellar assembly precursors by testing the prediction that relics would require prior assembly of the rod while assembly precursors would not. To abolish rod assembly, we developed an *S*. *putrefaciens* Δ*flhA* mutant incapable of assembling the rod due to disruption of the intrinsic flagellar type III secretion system. The mutant was nonmotile (Fig 3A), and of 68 cell poles imaged by ECT, no relic structures were seen (Figs 3B and S4), confirming that they require a functional flagellar type III secretion system despite being general secretory pathway (Sec) exported. Normal flagellar gene expression was confirmed by complementation with an *flhA* over-expression plasmid for which polar flagellation, motility, and relics were regained (Fig 3A and 3B).

Further confirming that flagellar partial structures are relics, we observed flagellar ejection “caught in the act” in one tomogram of wild-type *S*. *putrefaciens*, with a filament, hook, and rod structure adjacent to a flagellar partial structure (Fig 3C). The ejected filament retained both the hook and distal rod (Fig 3D), which correspond to the only structures missing from the motor; notably, the P-ring of the partial structure was already plugged with a plug protein (Fig 3E).

An orthogonal test for whether partial structures were relics or assembly intermediates was to inspect for anticipated interactions with the inner membrane because partial structures and intact flagella colocalize to the pole, suggesting a common localization mechanism. In the case of assembly intermediates, localization would likely be mediated via linkage to the inner membrane; in the case of relics, flagellar placement would have preceded ejection, and no connection to the inner membrane would be required. To test this, the distance between the inner- and outer-membrane disk was measured in fully formed motors as well as in partial structures. Periplasmic distances varied in both cases, as expected from previous work in *V*. *alginolyticus* [20]. Nevertheless, variation in membrane distance at the position of partial structures was far greater than at the intact flagella (13.9 nm and 7.9 nm range, respectively), suggesting that partial structures do not contain a connection with the inner membrane (Fig 3F).

These results, combined with the mixture of relic, rod, and hook genes interspersed throughout the same operon (Fig 3G), lead us to conclude that these structures are indeed relics of ejected flagellar motors made of an outer-membrane subset of flagellar proteins and not flagellar assembly intermediates.

### Motor and relic placement are not random and follow the same placement pattern

**Fig 4:**
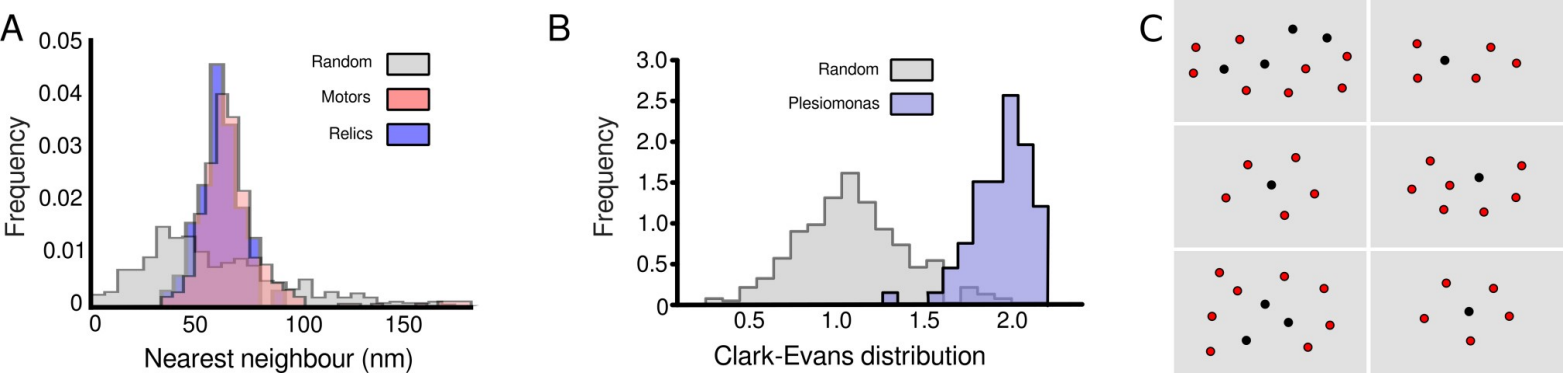
Relic positioning is the same as full flagella, indicating a common assembly placement. (A) Histograms for all nearest neighbors of relics (*N* = 114), motors (*N* = 424), and random synthesized data (*N* > 500). (B) Clark–Evans distribution of a random synthesized data set (*N* ≥ 500) compared to data from tomograms of *P*. *shigelloides* (*N* = 74). (C) 2D positions of relics (red circles) and motors (black circles) on six individual cells. Underlying data in S1 Data.

We observed that intact flagella and relics appear spaced on a grid at cell poles, suggesting that placement is not random and that both structures share a common grid. The nearest neighbor distances for all intact flagella and relics were determined and found to be on average 64 nm, approximately 20 nm greater than would be expected if distances were constrained by steric clashes of approximately 45-nm diameter C-rings. To assess whether the placement of both relics and intact flagella is the same, which would suggest a common mechanism for placement, histograms were plotted for all distances to nearest neighbors of relics, intact flagella, and random data (Fig 4A). The relics and intact flagella shared identical curves, with a much tighter distribution than the randomized data set, consistent with a common placement mechanism.

To quantify the dispersal of intact flagella and relic structures independent of the number of structures per cell, Clark–Evans distribution analysis [21] was calculated for each cell pole with more than four intact flagella or relics (*N* = 71) and compared to a synthesized random data set (Fig 4B). Values between 1 and 2.15 suggest a uniform grid, while values below 1 suggest clustering. A value close to 1 is a random dispersion. The approximately 500 synthesized random poles (constrained by the same number of structures and pole area as the real data) had a mean of 1, as expected. The *Plesiomonas* data set, however, had a mean close to 2. This shows that structures at the pole are placed in a nonrandom distribution in a grid-like arrangement and that the relics and intact flagella are part of the same grid (Fig 4C). Peritrichous flagella in *B*. *subtilis* have previously been shown to have a Clark–Evans ratio greater than 1 [22]. How new flagella avoid assembling beneath existing relics, however, is unclear.

### Ejection is triggered by the depletion of nutrients, not chemical or mechanical signals

Our initial results (Fig 1) indicated that an aspect of culture growth conditions triggers flagellar ejection. What, though, was the specific trigger? One possibility is that high cell density is physically detected by tangling of filaments. To test whether the trigger is mediated by mechanosensitivity of the load on the flagellar filament, a mutant lacking the polar flagellin FliC was made in *P*. *shigelloides* and imaged with ECT. Both motors with hooks as well as relics were observed in these cells, confirming that ejection does not require the flagellar filament (S5 Fig). This implies that mechanosensation of load on the motor by the filament is not the trigger for ejection. Furthermore, this observation is incompatible with mechanical shearing driving relic formation, as a short hook will be protected from shearing by the Reynolds shell around the bacterium, and unlikely to drive loss of the short hook.

**Fig 5:**
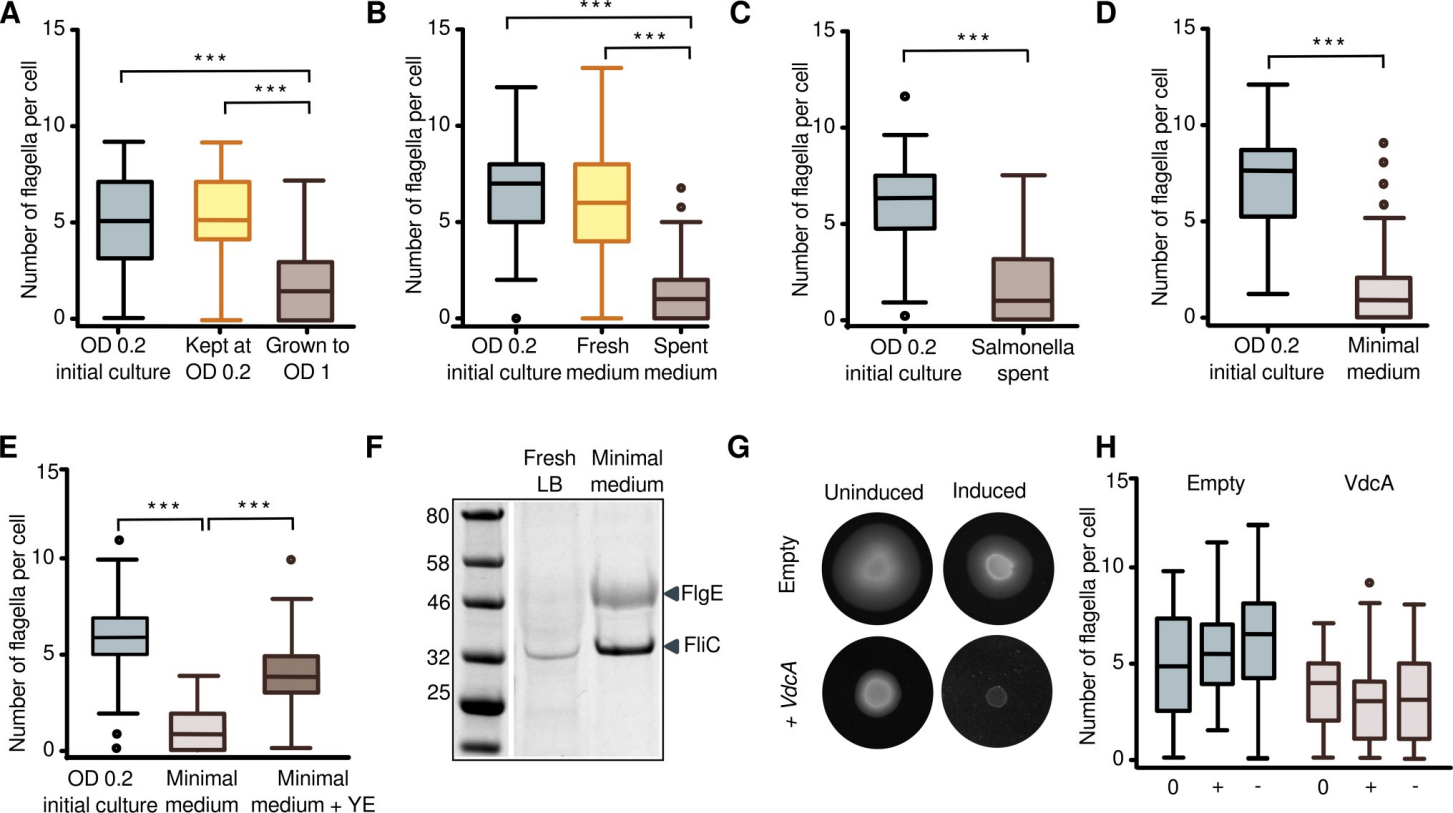
Ejection of the γ-proteobacterial flagellum is triggered by nutrient depletion. (A) Cells continually diluted to low cell density do not lose flagella (***P < 0.0001, one-way ANOVA). At least 65 cells were analyzed for each sample. (B) Low-OD cells moved from fresh LB to *P*. *shigelloides* spent medium lose flagella within one doubling time (***P < 0.0001, one-way ANOVA). The doubling rate was 2-fold lower for low cell density cells transferred to spent medium compared to those transferred to fresh LB. Ninety cells were analyzed from each sample. (C) Low-OD cells moved from fresh LB to *Salmonella* spent medium lose flagella within one doubling time (***P < 0.0001, unpaired *t* test). At least 75 cells were analyzed for each sample. (D) Cells eject flagella when transferred from LB to MOPS minimal medium (***P < 0.0001, unpaired *t* test). At least 60 cells were analyzed for each sample. (E) The ejection of flagella in minimal medium is rescued by the addition of yeast extract. More than 70 cells were imaged for each sample (***P < 0.0001, one-way ANOVA). (F) Supernatant of cells grown in minimal medium has detectable hook and more flagellin than the supernatant of cells grown in fresh LB. (G) Motility plates showing that cells harboring an over-expression plasmid for the c-di-GMP–synthesizing enzyme VdcA stop swimming but continue growing when induced. (H) Stopping swimming with increased levels of c-di-GMP is not due to ejection of flagella. 0 = prior to induction, - = not induced, + = induced. No significant difference was seen between the induced and not induced cells. Underlying data in S1 Data. c-di-GMP, cyclic di-GMP; FlgE, hook; FliC, filament; LB, Lysogeny Broth; OD, optical density.

To distinguish between cell density or nutrient depletion as the trigger for ejection, we tested whether cells continually diluted with fresh lysogeny broth (LB) retained their flagella. We compared the number of flagella on cells grown to an OD of 1 with that of cells grown over the same time period but continually diluted with fresh medium to maintain an optical density below 0.2. Undiluted bacteria had a mean of 1.7 flagella, while continually diluted bacteria had a mean of 5.3 flagella (Fig 5A), suggesting that the signal for ejection is intrinsic to the medium of old cells, not cell density.

To further confirm this, early growth–stage (low OD) cells were moved into spent medium and grown for one doubling time from OD 0.2 to 0.4 (doubling time in fresh LB = 19 minutes and in spent medium = 38 minutes). Within a single cell cycle, multiple flagella were lost, with mean number of flagella dropping from 6.7 to 1.2. A control culture grown in fresh LB and negatively stained at the same optical densities retained all flagella (Fig 5B). This result suggests that the trigger for flagellar ejection is in the supernatant of cells that have lost flagella.

To distinguish whether the signal in the supernatant is a species-specific chemical secreted by late growth–stage cells (high OD), or depletion of a medium component, *P*. *shigelloides* cells were grown to low OD and transferred to spent supernatant from *Salmonella* grown to stationary phase. As with *P*. *shigelloides* spent medium, within one cell division in *Salmonella* spent medium, *P*. *shigelloides* cells lost multiple flagella (mean number of flagella went from 6.2 to 1.6) (Fig 5C), suggesting that the signal is depletion, not presence of a signaling molecule. To confirm this, we moved early growth–stage (low OD) cells from LB into MOPS minimal medium (Fig 5D). Cells stopped swimming within an hour, and within one doubling time, the cells had lost most of their flagella as counted by negative-stain electron microscopy, with a drop in mean number of flagella from 5.8 to 1.6. We further confirmed that the ejection of flagella can be rescued by adding yeast extract to the minimal medium (Fig 5E). Together, these results confirm that lack of nutrients is a necessary and sufficient signal for ejection of flagella and entry into a nonmotile state.

These results are best compatible with an active ejection mechanism. To further test whether ejection is active, we distinguished between passive random filament breakage and determinate ejection at the base of the hook. We grew cells to low OD in LB before washing and resuspending them into either fresh LB or MOPS minimal medium and left them to grow for one doubling time. Supernatant from both cultures was PEG precipitated and run on an SDS-PAGE gel (Fig 5F). Not only was the amount of shed flagellin (FliC) in supernatant from cells grown in minimal medium substantially greater than those grown in fresh LB, but also, supernatant from cells moved into fresh LB had no hook protein, whereas the supernatant from cells moved into minimal medium had substantial amounts of hook protein. The flagellin from the fresh LB sample may therefore result from a small amount of random filament breakage, while minimal medium triggers a distinct ejection of flagellar filaments and hooks from the base of the hook. This confirms the substantially increased rate of flagellar ejection under nutrient depletion conditions and further supports an active ejection mechanism, as opposed to passive breakage.

In many bacteria, the transition from motile to sessile states is mediated by concentration changes of the intracellular second messenger c-di-GMP [23]. To determine whether flagellar ejection is mediated by c-di-GMP signaling, we overexpressed the c-di-GMP-synthesizing diguanylate-cyclase VdcA from *V*. *cholerae* in *P*. *shigelloides* cells and monitored motility and flagellar number (Fig 5G and 5H). As expected, motility was abolished in *P*. *shigelloides* cells overexpressing VdcA but not in wild-type (WT) cells harboring an empty vector and grown under the same conditions. Surprisingly, however, when we counted flagella on cells induced to overexpress VdcA, we saw no decrease in number of flagella (Fig 5H). This result demonstrates that flagellar ejection in *P*. *shigelloides* is not under c-di-GMP control.

We attempted to identify the molecular mechanism underlying flagellar release and ejection. We used transposon insertions to screen for mutants incapable of detecting nutrient depletion, reasoning that these would retain motility at high cell densities, and set up suppressor screens on motility plates made from minimal medium or spent medium to identify motility flares but identified no candidates (results not shown). We speculate that the trigger mechanism is redundant, requiring simultaneous mutation of multiple targets. We also tested the effect of uncoupling the proton gradient across the inner membrane using CCCP; although motility was lost, flagella were not ejected. Based on the analogy of ClpA-mediated flagellar ejection by *C*. *crescentus* [24], we tested whether ClpA or ClpX were responsible for flagellar ejection. Flagellar ejection rates were not reduced, however, in either *clpA* or *clpX* deletion mutants. The molecular mechanism of flagellar ejection, therefore, remains unclear.

## Discussion

Here, we describe a new mechanism of switching from motile to nonmotile states in the polarly flagellated γ-proteobacteria. To achieve this, we used electron microscopy to analyze the structural changes in species that showed characteristic decreases in swimming speeds when grown to high cell density. We found that these bacteria respond to nutrient depletion by shifting from motile to nonmotile state by ejecting their flagellar hook, distal rod, and filament into the supernatant and found the rate of loss is increased upon nutrient depletion and cannot be attributed to mechanical or arbitrary mechanisms. Using ECT, we showed that the relics of these ejected flagella are retained by the cell and remain at old cell poles. Subtomogram averaging revealed that relics were plugged by a novel protein complex that likely functions to prevent periplasmic leakage. We further showed that plugged relics are found in all of the polarly flagellated γ-proteobacterial species imaged to date: *P*. *shigelloides*, *V*. *cholerae*, *V*. *fischeri*, *S*. *putrefaciens*, and *P*. *aeruginosa*.

Our results, together with previous data, show that the outer-membrane partial flagellar structures are relics and not assembly intermediates awaiting insertion of a rod, hook, and filament. γ-proteobacterial polar flagella loss correlates with recovery of flagella with hooks from the supernatant; a decrease in swimming speed, flagella per cell, and flagella in the population (Fig 1); and appearance of more relic structures (Table 1). Appearance of multiple relics at a single pole of *V*. *cholerae*, a bacterium that only ever assembles exactly one polar flagellum, is incompatible with controlled assembly of a single polar flagellum regulated in the initial stages of flagellar formation [25] but consistent with relics arising from multiple rounds of flagellar synthesis and ejection. Furthermore, P- and L-rings, which form the core of the relics, require a distal rod for assembly [18] and multiple P-rings assemble around polyrods, showing that the rod, and not the outer membrane, triggers P-/L-ring formation [26,27], arguing that the P- and L-ring relic core cannot assemble without the rod. Indeed, recent work in *V*. *alginolyticus*, a close relative of *V*. *fischeri* and *V*. *cholerae*, suggests that the H-ring is prerequisite in outer-membrane penetration in bacteria with sheathed flagella [12]. Furthermore, the selective assembly of relic components FlgH, FlgI, FlgO, FlgP, FlgT, MotX, and MotY is incompatible with the structure of their operon, in which they are interspersed with flagellar rod and hook genes (Fig 3G). (Although we cannot rule out the different mechanisms of secretion—i.e., Sec and the flagellar type III secretion system—segregating relic structures from axial structures actively during assembly, such an operon structure would likely face considerable selective pressure to fragment into separate modules representing discrete, independent structural components; operon structure is well-known as a hallmark of structural coassembly [28,29].) Also, the range in periplasmic distance at relics is far greater than at full motors, suggesting no link between the relics and the inner membrane, and motors and relics are positioned on a common grid, suggesting a common assembly mechanism (Fig 4), likely from the cytoplasm, despite absence of attachment to the inner membrane. Finally, in a T3S-deficient strain (Δ*flhA*), no relic structures were seen, a final confirmation that these structures cannot be assembly intermediates (Figs 3 and S4).

### Use of a plug protein indicates a specific cellular response

A striking feature of relics is the addition of a “plug” that is filling the space usually occupied by the distal rod. A targeted response to the ejection of the flagellum in the form of a plug is significant, as this plug may prevent leakage of cellular components. Plug placement is likely rapid, as a tomogram of *S*. *putrefaciens* caught immediately after ejection has a partial motor that already contains the plug protein, and we observed no relics without a plug in any of the organisms. The plug density in the relic subtomogram average is large, and no density in the subtomogram average of the full motor can account for this (if it were a gate mechanism as seen in the injectisome secretin [30]). We therefore believe that this is a unique protein not already in the flagellar motor and not secreted via the flagellar type III secretion system.

We were able to partially purify relics but were unsuccessful in identifying the plug protein using mass spectrometry approaches, likely due to low copy number of relics. Although it is possible that we were unsuccessful identifying the plug because it is a known flagellar protein that has undergone a conformational change to become the plug, analysis of subtomogram averages argues against this possibility, as all other densities remain intact and retain their conformations. Another possibility is that the plug is a flagellar protein that does not form part of the motor. Possible candidates include one of the rod proteins, the rod cap FlgJ, or the P-ring assembly factor FlgA. While we cannot rule out these possibilities, such a conformational change would be unprecedented, and we favor the speculation that the plug is composed of a yet-to-be-discovered enigmatic protein.

The alternative strategy to prevent periplasmic leakage would be disassembly of the entire relic structure and resealing of the outer membrane, requiring evolution of an intricate machinery for proteolysis of all components. Evolution is a tinkerer [31], and plugging the hole is a considerably more trivial solution than orchestrated disassembly, particularly given that retaining plugged relic structures is unlikely to be detrimental to the cell.

We speculate that in the relatively well-studied case of *C*. *crescentus*, flagellar ejection of a similar plugging mechanism may occur [32]. Although an active mechanism for rearrangement of the outer membrane after flagellar ejection is likely to exist to prevent leakage of periplasmic components out of the cell, at present it is unclear what this might be and what the fate of the outer-membrane components is after ejection. The *C*. *crescentus* flagellar motor, however, does not contain large outer-membrane disks [2], so the visualization of a *Caulobacter* relic, made up of only the P and L-rings, would prove difficult with ECT.

### Mechanism of flagellar ejection

Our results demonstrate that flagellar ejection is active and triggered by nutrient depletion. Increased rate of flagellar loss during nutrient depletion, determinate ejection at the base of the hook only under nutrient depletion, ejection even of hooks regardless of presence of the filament, and presence of a plug combine to argue against passive, arbitrary flagellar loss. Indeed, we routinely monitor γ-proteobacterial motility in more viscous media, yet do not observe abolished swimming motility due to viscosity changes, as seen in a *Salmonella* FliF mutant that readily loses its distal rod, hook, and filament when swarming or in higher viscosity medium [33,34].

The proteins that drive flagellar ejection remain to be determined. In *C*. *crescentus*, the protease ClpAP is associated with degradation of FliF at the time of flagellar ejection, although it is unclear whether this is causal or caused by ejection [35]. In *S*. *putrefaciens*, however, we found that neither ClpA nor ClpX were required for ejection.

### The selective benefit of flagellar ejection

The γ-proteobacteria with Na^+^-driven motors eject their flagella and enter a nonmotile state, unlike *Salmonella*. As ejection occurs in nutrient limited environments, this may be a mechanism for bacterial cultures to conserve energy by cutting back on their more extravagant energy expenditures such as flagellar motility.

Why polarly flagellated γ-proteobacteria eject filaments under nutrient depletion, whereas *Salmonella* and *E*. *coli* simply cease assembly (leading to a decrease in flagellar number over time due to dilution), is unclear. The difference may be due to differences in placement of flagella: being peritrichous, *Salmonella* and *E*. *coli* lose flagella by dilution as the cell elongates. With the polar flagellates, motors are polar and therefore not diluted as the cell elongates. Rather, one daughter cell inherits an entire set of flagella. Ejection, therefore, is a drastic but potentially necessary survival mechanism for those daughter cells that inherit the burden of old, undiluted, fully-flagellated poles. *V*. *fischeri* has previously been shown to lose flagella upon colonization of the squid light cavity and regrow them when exposed to fresh seawater [36], although how this observation generalizes over other γ-proteobacteria is unclear. Flagellation levels have been shown to decrease as bacteria enter stationary phase. We suggest that flagellar ejection is a mechanism to reduce the number of flagella on a cell pole. Not only do active flagella require energy for torque generation, but flagellar assembly is a constant process and a constant drain on cell energy [37]. Though drastic, decreasing the number of flagella by ejection would be an effective energy conservation measure. Furthermore, this mechanism may be specific for polar flagella: there is no evidence that lateral flagella, assembled by species including *P*. *shigelloides*, are ejected; such flagella will be diluted as with the peritrichous flagella of *Salmonella* and *E*. *coli*. It is also noteworthy that loss of flagella will inevitably reduce the Reynolds shell around the bacterium, effectively making the bacterium smaller, although the selective benefit of this is unclear.

### Model for assembly and ejection of the Na^+^-driven polar flagellum

**Fig 6:**
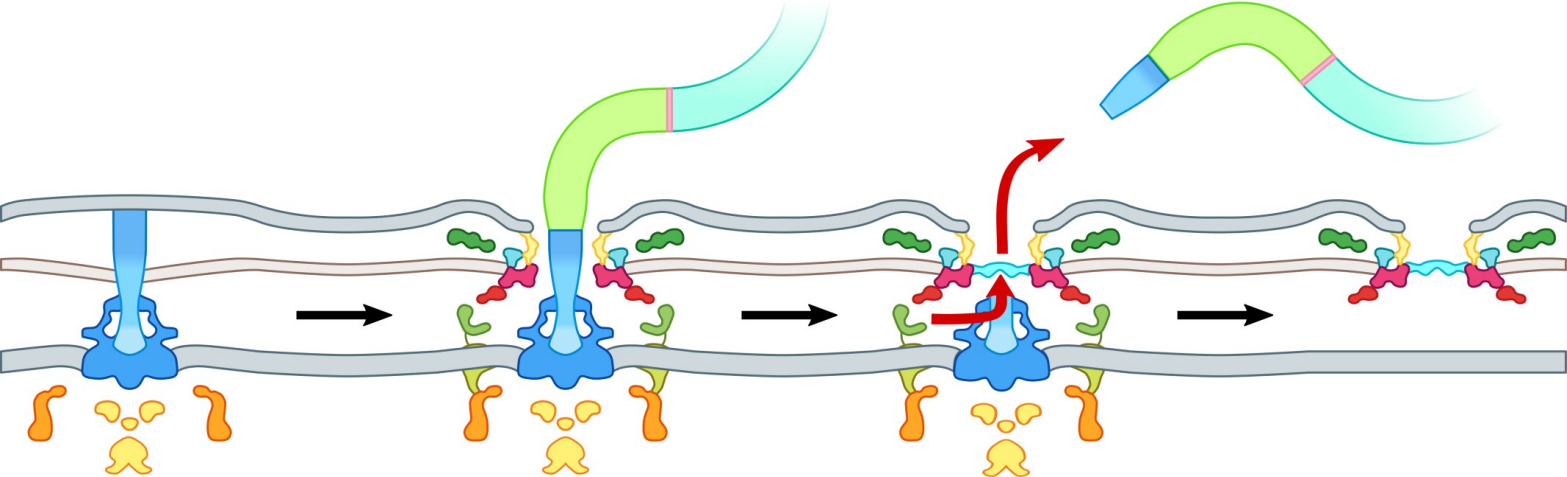
A model for γ-proteobacterial flagellar ejection. Schematic for the assembly and ejection of the γ-proteobacterial polar flagellum. Colors match genes in Fig 3G and structures in Fig 2C and 2E.

Our results enable interpretation of the multiple states of the flagellum caught in tomograms (Fig 2B). We propose a model for the steps of assembly and ejection of the polar γ-proteobacterial flagellum (Fig 6). After assembly of the MS-ring, C-ring, and flagellar type III secretion system, the rod is assembled. Recent work in *Salmonella* showed that polymerization of the rod is stopped by hitting the outer membrane [38]. In subtomograms of this stage, the rod appears to hit the outer membrane perpendicular and push on it, forming a bulge in the outer membrane. We believe that the same mechanism for stopping rod polymerization in the enteric motors is found in polar flagellar motors. Once the P- and L- rings form around the rod, FlgO, FlgP, FlgT, MotX, and MotY can assemble on this platform, and the hook and filament can be assembled through the channel in the outer membrane formed by the L-ring. Upon nutrient depletion, however, the filament, hook, and distal rod are released and a plug protein fills in the leftover gap. The inner-membrane and C-ring components are finally cleared away. The ejection of flagella in nutrient depleted environments and subsequent rebuilding when exposed to fresh nutrients is a novel mechanism for the transition between a planktonic and a sessile state shown here to be widespread amongst the Na^+^-driven polar flagellated γ-proteobacteria.

## Methods

### Cell growth

Strains and plasmids used in this study are tabulated in Table 2.

**Table 2:** Strains and plasmids used in this study.

Strain or Plasmid	Source
*Plesiomonas shigelloides* ATCC 14029 WT	DSMZ (DSM-8224)
*Shewanella putrefaciens* CN-32 WT	[40]
*Vibrio fischeri* ES114 WT	Edward G. Ruby (University of Hawai‘i at Mānoa)
*Vibrio cholerae* N16961 WT	Simon Ringgaard (MPI Marburg)
*Pseudomonas aeruginosa* PAOI WT	Kylie Watts (Loma Linda University)
*E*.*coli* WM3064	W. Metcalf, (University of IIlinois)
*S*. *putrefaciens* CN-32 Δ*flhA*	This study
*S*. *putrefaciens* CN-32 motX-mCherry-His	[41]
pBTOK	[42]
pBTOK-VdcA	This study
pBTOK-FlhA	This study
*S*. *putrefaciens* CN-32 Δ*clpA*	This study
*S*. *putrefaciens* CN-32 Δ*clpX*	This study
*Plesiomonas shigelloides* Δ*fliC*	This study

**Abbreviations:** ATCC, American Type Culture Collection; DSMZ, Deutsche Sammlung von Mikroorganismen und Zellkulturen; WT wild-type.

*P*. *shigelloides* was grown in LB (10 g/L tryptone, 10 g/L NaCl, 5 g/L yeast extract) at 37°C, 200 RPM overnight in 14-ml aeration tubes. The next day, cultures were diluted 1:100. Cells were grown to an OD of 0.2 unless specified.

*S*. *putrefaciens* CN-32 was grown in LB (10 g/L tryptone, 10 g/L NaCl, 5 g/L yeast extract) at 30°C, 200 RPM overnight in 14-ml aeration tubes. The next day, cultures were diluted 1:100 for experiments. Cells were grown to an OD of 0.5.

*V*. *fischeri* was grown in LBS + MgSO_4_ (10 g tryptone, 5 g yeast extract, 20 g NaCl, 8.62 g MgSO_4_) at 30°C, 200 RPM overnight in 14-ml aeration tubes. The next day, cultures were diluted 1:100 for experiments. Cells were grown to an OD of 0.3.

*V*. *cholerae* N16961 was grown in LB for 18 h, harvested by centrifugation, and washed twice with sterile filtered and autoclaved environmental sea water before introduced into a 50-ml sea water microcosm at an OD_600_ 2.5. Microcosms were kept at low agitation at 4°C for one month prior to imaging.

*P*. *aeruginosa* PAOI persister cells were obtained from overnight stationary phase culture treated with carbonyl cyanide *m*-chlorophenylhydrazone (CCCP) 200 μg/ml for 3 h in LB, followed by exposure to ciprofloxacin 5 μg/ml for 3 h in sodium-phosphate buffer (10 mM, pH 7.4), as described previously [39].

### Swimming speed videos

To measure bacterial swimming speeds at different cell densities, cells were grown as stated above and diluted in LB or LBS to an OD_600_ of 0.2 immediately prior to imaging. Cells were imaged at 100x oil immersion on a MT4000 series light microscope (Meiji Techno) with a DMK23G618 digital camera connected to IC Capture software. Bacterial cells were tracked for 10 s using Fiji [43] and converted to swimming velocities using custom scripts. Three videos were taken at each time point, providing over 1,000 traces, and this was done in triplicate. Error bars indicate standard error.

### Negative-stain EM

3 μl of cells or flagella were placed on glow-discharged continuous-carbon grids (Taab Laboratory Equipment Ltd.). The grids were stained with 2% uranyl acetate and stored at room temperature prior to imaging. Negative-stain micrographs were collected on either an FEI Tecnai G2 Spirit BioTWIN (tungsten filament) using an Eagle CCD camera or a Technai T12 TWIN (LaB_6_) using a TVIPS F216 CCD camera. Images were viewed using 3DMOD [44,45], and number of flagella per cell (or number of filaments with hooks) was counted manually.

### Purification of relics

An *S*. *putrefacien*s MotX-mCherry-HIS strain was grown overnight at 30°C. The cultures were diluted 1:100 into 5 L of LB and grown into stationary phase. Cells were pelleted and resuspending into MOPS minimal media and grown for a further 2 h to ensure flagella were ejected. Cells were pelleted and resuspended into 150 mM Tris pH 7.5, 150 mM NaCl, 10% sucrose, 10% glycerol, DNase, and EDTA-free protease inhibitors. Cells were passed once through a cell disrupter (27 kpsi) and centrifuged at 15,000 x g for 15 min to remove unlysed cells. The supernatant was ultracentrifuged for 1 h and the membrane pellet was washed in 150 mM Tris pH 7.5, 150 mM NaCl, 10% Sucrose, and 10% glycerol before further centrifugation. Membrane pellets were flash frozen in 150 mM Tris pH 7.5, 150 mM NaCl, 10% Sucrose, and 10% glycerol. Thawed membranes were solubilized in 1% DDM for 1 h at 4°C before dilution in 150 mM Tris pH 7.5, 150 mM NaCl, 10% Sucrose, 10% glycerol, 0.03% DDM, and 20 mM imidazole. Soluble fraction was loaded onto a His-trap and washed with buffer up to 100 mM imidazole. Relics were eluted with 300 mM imidazole.

### 2D classification of relic particles

Negative-stain grids were prepared as above. Images were collected on a Technai T12 TWIN (LaB_6_) using a TVIPS F216 CCD camera with a pixel size of 2.586 Å. Processing was done within Scipion 1.2 [46], particles were picked using Xmipp3 [47], and classification was done using Relion [48].

### PEG precipitation to recover ejected flagella

Cells were spun down three times for 30 min at 3220 × *g*, and supernatant was carefully decanted. The supernatant was assessed with light microscopy to make sure no cells remained. We subsequently added 3% PEG 20,000 to the supernatant and stirred at RT for >1 h or at 4°C overnight. Flagella were recovered by centrifugation at 3220 × *g* for 20 min.

### SDS-PAGE gel electrophoresis of recovered flagella

After PEG precipitation, flagella were boiled in LDS and run on a 4%–12% Bis-Tris gel (Thermo-Fisher, Hillsboro, OR, United States of America) and stained with InstantBlue (Sigma-Aldrich, Taufkirchen, Germany).

### Mean number of flagella in a population

CFU at each OD was calculated by plating cells on LB or LBS agar and counting the number of colonies. The mean number of flagella at each time point from negative-stain images was multiplied by the total number of cells in the population.

For all experiments, at least three biological replicates with 30–50 cells per time point in each were imaged.

### Electron cryo-tomography sample preparation

For *P*. *shigelloides*, *S*. *putrefaciens*, and *V*. *fischeri*, cells were pelleted by centrifugation at 3824 × *g* for 5 min and resuspended to an OD_600_ of 11. Cells were mixed with gold fiducials coated in BSA, and 3 μl of the cell fiducial mixture was applied to freshly glow discharged R2/2, 200 mesh gold Ultrafoil or copper Quantifoil R2/2 grids (Quantifoil Micro Tools GmbH, Germany). A Vitrobot Mark IV was used to freeze grids in an ethane/propane cryogen at 100% humidity.

*V*. *cholerae* and *P*. *aeruginosa* cells from cultures were directly mixed with protein A-treated 10-nm colloidal gold solution (Cell Microscopy Core, Utrecht University, Utrecht, the Netherlands). After mixing, aliquots of 3 μl were applied to freshly plasma-cleaned R2/2, 200 mesh copper Quantifoil grids (Quantifoil Micro Tools GmbH, Germany). Plunge freezing was carried out in liquid ethane using a Leica EMGP (Leica microsystems, Wetzlar, Germany). During 1 s blotting time, the blotting chamber was set at room temperature (20°C) with 95% humidity.

### ECT data acquisition

Tomograms of *Plesiomonas*, *Shewanella*, and *V*. *fischeri* were collected on an FEI F20 with a Falcon II detector (Thermo Fisher Scientific [formerly FEI], Hillsboro, OR, USA). A total fluence of 120e^−^/ Å^2^ was used and a defocus of −3 to −4 μm. Tomograms were collected at a tilt range of ±53 degrees with 3° tilt increments and a pixel size of 8.28 Å.

Tomograms of *V*. *cholerae* and *P*. *aeruginosa* were performed on a Titan Krios transmission electron microscope (Thermo Fisher Scientific [formerly FEI], Hillsboro, OR, USA) operating at 300 kV. Tomograms were recorded with a Gatan K2 Summit direct electron detector (Gatan, Pleasanton, CA) equipped with a GIF-quantum energy filter (Gatan), operating with a slit width of 20 eV. Images of *V*. *cholerae* and *P*. *aeruginosa* were taken at a nominal magnification of 42,000 and 26,000, respectively, which corresponded to a pixel size of 3.513 Å and 5.442 Å. Using UCSFtomo software, all tilt series were collected using a bidirectional tilt scheme of ±53 degrees (*V*. *cholerae*) and of ±60 degrees (*P*. *aeruginosa*), respectively. Defocus was set to −8 μm.

The *P*. *shigelloides* data set for subtomogram averages was collected on a Titan Krios operating at 300 kV with a post-column GIF using a 20 eV slit and a Gatan K2 summit (Gatan, Pleasanton, CA), collecting 4 frames per exposure. A pixel size of 2.713 Å with a total fluence of 54 e^−^/A2, and a defocus range between −3 and −5.5 μm was used. The bi-directional tilt series was collected using FEI’s Tomo4 (Thermo Fisher Scientific [formerly FEI], Hillsboro, OR, USA) with a tilt range of ±53 degrees and a tilt increment of 3°.

### Tomogram reconstruction

For the *P*. *shigelloides* data set, frames were aligned using UnBlur [49]. Tomoctf [50] was used to estimate CTF parameters (tomoctffind) and correct stacks (ctfcorrectstack). Fiducials were picked from CTF-corrected stacks and tracked in IMOD [44,45]. The aligned, CTF-corrected stacks and fiducial models were used with Tomo3d to reconstruct using SIRT or WBP [51,52].

For *V*. *fischeri* and *S*. *putrefaciens*, tomograms were reconstructed using RAPTOR [53].

For *V*. *cholerae* and *P*. *aeruginosa*, drift correction and fiducial tracking–based tilt series alignment of tomograms were done using the IMOD software package [44,45]. Tomograms were reconstructed using SIRT.

### Subtomogram averaging

Subtomograms were picked manually from 2x binned tomograms. Template-free alignment was carried out in PEET by superimposing all manually picked subtomograms, allowing no shifts or rotations for an initial reference. Initial alignment and averaging was achieved using binned subtomograms. Unbinned subtomograms were used for further refinement. As the rod length varied in the full motor, subtomogram averaging was carried out twice with custom masks applied to either the top ring structures or the cytoplasmic structures. As clear 13-fold symmetry was observed in the full motor (S2 Fig), 13-fold rotational averaging was applied using custom scripts. The two individual averages for the top and bottom segments were then merged into a composite structure as in [9]. For comparisons, 13-fold rotational averaging was applied to the relic structure. FSC curves were determined from the final, unsymmetrized averages using PEET’s calcFSC (S2 Fig).

### Segmentation

Semi-automatic segmentation of the membranes was performed in IMOD [44,45] using the Sculpt drawing tool followed by the linear interpolator.

### Periplasmic distance measurements

Contours were drawn between the inner membrane and the first ring of the outer-membrane disks manually in 3DMOD (IMOD package [44,45]), and distances were measured.

### Clark–Evans distribution analysis

Clark–Evans ratios were calculated per cell with more than four flagella (*N* = 70) [21]. To calculate the Clark–Evans distribution, the mean nearest neighbor distance was divided by half the square root of the mean cell pole density. The mean nearest neighbor distance was calculated using custom scripts, and the cell pole area density was calculated for each pole. The area of each pole was calculated by adding the diameter of one C-ring (45 nm) to the maximum X and Y coordinates of any motor or relic structure, and these coordinates were used to calculate the area of an ellipse. To calculate the mean cell pole density, each pole area was divided by the number of structures at that pole.

### Nutrient depletion assay

Cells grown overnight were removed from the supernatant by centrifugation. The supernatant was subsequently filtered using a 0.2-μm filter. *Plesiomonas* cells grown to low OD_600_ (0.2) were spun down and resuspended in the filtered, spent medium. Negative-stain grids were made prior to resuspension and after one doubling time in spent medium.

### Minimal medium assay

*Plesiomonas* cells grown to low OD_600_ (OD 0.2) were spun down and resuspended in MOPS minimal medium (40 mM MOPS pH 7.4, 4 mM Tricine, 0.1 mM FeSO_4_·7H_2_O, 9.5 mM NH_4_Cl, 0.28 mM KCl, 0.53 mM MgCl_2_·6H_2_O, 50 mM NaCl, 10 mM KH_2_PO_4_, 0.005% MgSO_4_, 0.0005% MnCl_2_·4H_2_O, 0.0005% FeCl_3_, and 0.5% glucose). Cells were examined by light microscopy after 1 h to see if they were swimming. Negative-stain grids were made prior to resuspension and after one doubling time in spent medium.

### Strain construction

DNA manipulations were carried out using appropriate kits (VWR International GmbH, Darmstadt, Germany) and enzymes (Thermo Fisher, St Leon‐Rot, Germany; New England Biolabs, MA, USA). Plasmids were constructed using standard restriction-ligation techniques or by the Gibson assembly method [54].

To generate deletion mutants in *S*. *putrefaciens* and *P*. *shigelloides*, two 500–600-bp flanking regions upstream and downstream of the gene of interest were amplified and fused together using appropriate primers (for *SpclpA* deletion: GCG AAT TCG TGG ATC CAG AT GAT AGT AAA ACC TGT ATA GAC GGG, GTT AAG CCT T CAG ATC TTT GTT CAG CAT AAG CAC, CAA AGA TCT G AAG GCT TAA CTC GGC CTA AAG G, GCC AAG CTT CTC TGC AGG AT GGA TAT CAA ATA GAT GCA AAC CG; for *SpclpX* deletion: GCG AAT TCG TGG ATC CAG AT CAT GGC CAA TTT GAT TGT GGC G, ATT GTT CGC C GTC GCC CAT TAA TTA CCT CAT TTG, AAT GGG CGA C GGC GAA CAA TAA TTG TAC TGA TTG C, GCC AAG CTT CTC TGC AGG AT ACC TTC AAA CTG CCC AAT AGC G; for *SpflhA* deletion: GTA GCT AGC ACG TAG TGA TGC TAA TTC ATC ACG, TGT TAA AGC GGT CGG CCA GTA GAG GAC G, ACT GGC CGA CCG CTT TAA CAT CCA TTC ACT ATT AC, TCC GGG CCC GGA CGC GTT CGA CAA ATG CA; for *PsfliC* deletion: TCT CTG CAG GAT ATC CAA CAA ACT GGT GAC CGA AAG AGC C, TAA CCC AGA ATA GCC ATG CTT GTA ATC TCC GTT, GGC TAT TCT GGG TTA ATT CTT TCT CAG CCA AG, GAA TTC GTG GAT CCA ATA TCT CGG TGC GTT ATA CTT CGG T). Delivery into *S*. *putrefaciens* and *P*. *shigelloides* occurred by conjugation from *E*.* coli* WM3064 and markerless sequential crossover into the native site of the genes using the suicide plasmid pNPTS‐138‐R6K [55]. Correct deletions were verified by polymerase chain reaction (PCR) using appropriate primers (for *SpclpA* deletion: CCA TTT AAC AGT TCC GCT TGC C, TGA TGA AGC CCA TCG TTC ACT C; for *SpclpX* deletion: CTA AGG GTG AAC GCT CAT ACG A, AAG AGG TTA AGA CTT CTG GCG G; for *SpflhA* deletion: TAG ACT TTG GGG TAG TGC TCG, CAG GGA ATG TTC ACC ATG CG; for *PsfliC* deletion: TTC ATT TCA TCA CGT GAA TAA ATG ATG ATT TTT TTA GGG T, ACA CTA TTG CTC AGC GAT AAC GAA TAA ATG ATG TAG GAA G).

Inducible expression of proteins in *P*. *shigelloides* was carried out using the pBTOK vector [42]. The oligonucleotide pair CGC TCT AGA AGG AGG GCA AAT GTG ATG ACA ACT GAA GAT TTC AAA A and TCC GGG CCC TTA GAG CGG CAT GAC TCG ATT G was used to amplify the gene encoding the diguanylate cyclase VdcA (VCA 0956) from *V*. *cholerae*. The oligonucleotide pair AAT TCA GTC GGG CGA CAT CAC CAT CAC CAT CAC TAA GCT TGG ACT CCT GTT GAT and ATC AAC AGG AGT CCA AGC TTA GTG ATG GTG ATG GTG ATG TCG CCC GAC TGA ATT was used to amplify the gene encoding FlhA from *S*. *putrefaciens*. Subsequent delivery to *P*. *shigelloides* and *S*. *putrefaciens* was achieved by conjugation from *E*.* coli* WM3064.

### Soft-agar plates

*P*. *shigelloides* cells harboring either empty pBTOK vector or expressing the diguanylate cyclase VdcA were stabbed into soft-agar plates (10 g/L Bacto-Tryptone, 5 g/L NaCl, 3 g/L Bacto-Agar) containing 50 μg/ml kanamycin and either no inducer or 2 ng/ml anhydrotetracycline and grown at 37°C for 6 h.

### c-di-GMP overproduction

*P*. *shigelloides* cells harboring either empty pBTOK vector or expressing the diguanylate cyclase VdcA were grown with 50 μg/ml kanamycin to OD_600_ 0.2. Cells were then split in two and either not induced or induced with 2 ng/ml anhydrotetracycline until an OD_600_ of 0.4 was reached. Negative-stain grids were made prior to and after induction.

## Supporting information

S1 Fig Lysed *P*. *shigelloides* cells show multiple flagellar relics at the pole. (A) Slice through a tomogram with red arrows highlighting relic structures. (B) A different slice from the same tomogram as shown in (A) showing the top view of more relic structures.
(TIFF)

S2 Fig Analysis of subtomogram averages. FSC curves of subtomogram averages and subtomogram averages of the *P*. *shigelloides* motor (top panel) and relic structure (bottom panel) before and after applying symmetry. Resolution at a 0.5 threshold of the unsymmetrized motor structure is approximately 3.3 nm, and the unsymmetrized relic is approximately 4.7 nm. The two bottom slices of the top panel show the 13-fold symmetry through the T-ring planes in the top central slices. FSC, Fourier Shell Correlation.
(TIFF)

S3 Fig Relic structures are made of the same proteins as flagellar motors and can be visualised by negative-stain EM. (A) Example particles extracted from negative-stain EM images of relic structures isolated using affinity purification of a *S*. *putrefaciens* MotX-His strain. (B) Example 2D class average of relic structures showing concentric rings. (C) Slice through a single tomogram of *P*. *shigelloides* showing concentric rings. (D) Slice (50 voxels thick) through the relic subtomogram average of *P*. *shigelloides*. (E) Slice (50 voxels thick) through the motor subtomogram average of *P*. *shigelloides*. EM, electron microscopy.
(TIFF)

S4 Fig Partial flagellar motors are relics of old motors and are not formed in mutants incapable of assembling rods. Slices through six representative tomograms of Δ*flhA* cells. No relics were seen at the poles of any of the 68 cells imaged. Red arrows indicate chemoreceptor arrays.
(TIFF)

S5 Fig Flagellar filaments are not required for flagellar ejection. (A) Slice through a tomogram of *P*. *shigelloides* Δ*fliC* showing intact motors with hooks but no filament. (B) Slice through a tomogram of *P*. *shigelloides* Δ*fliC* showing multiple relics (red arrows).
(TIFF)

S6 Fig Placement of motors and relics in 3D. The 3D placement of relics and full flagellar motors on the pole of a representative cell. Red arrows point to relics, green flagellar filaments indicate full motors.
(TIFF)

S1 Data Underlying data for Figs 1A, 1C, 1D, 3F, 4A, 4B, 5A, 5B, 5C, 5D, 5E and 5H. (XLSX)

## Abbreviations

CCCP: Carbonyl cyanide *m*-chlorophenyl hydrazone
c-di-GMP: cyclic di-GMP
CFU: colony-forming unit
ECT: electron cryo-tomography
EM: electron microscopy
FSC: Fourier Shell Correlation
OD: optical density
SEC: general secretory pathway
WT: wild-type
