# Supplementary figures and images for: γ-proteobacteria eject their polar flagella under nutrient depletion, retaining flagellar motor relic structures
Source: PLoS Biol. 2019 Mar 19;17(3):e3000165. doi: 10.1371/journal.pbio.3000165 (PMC6424402; doi:10.1371/journal.pbio.3000165)

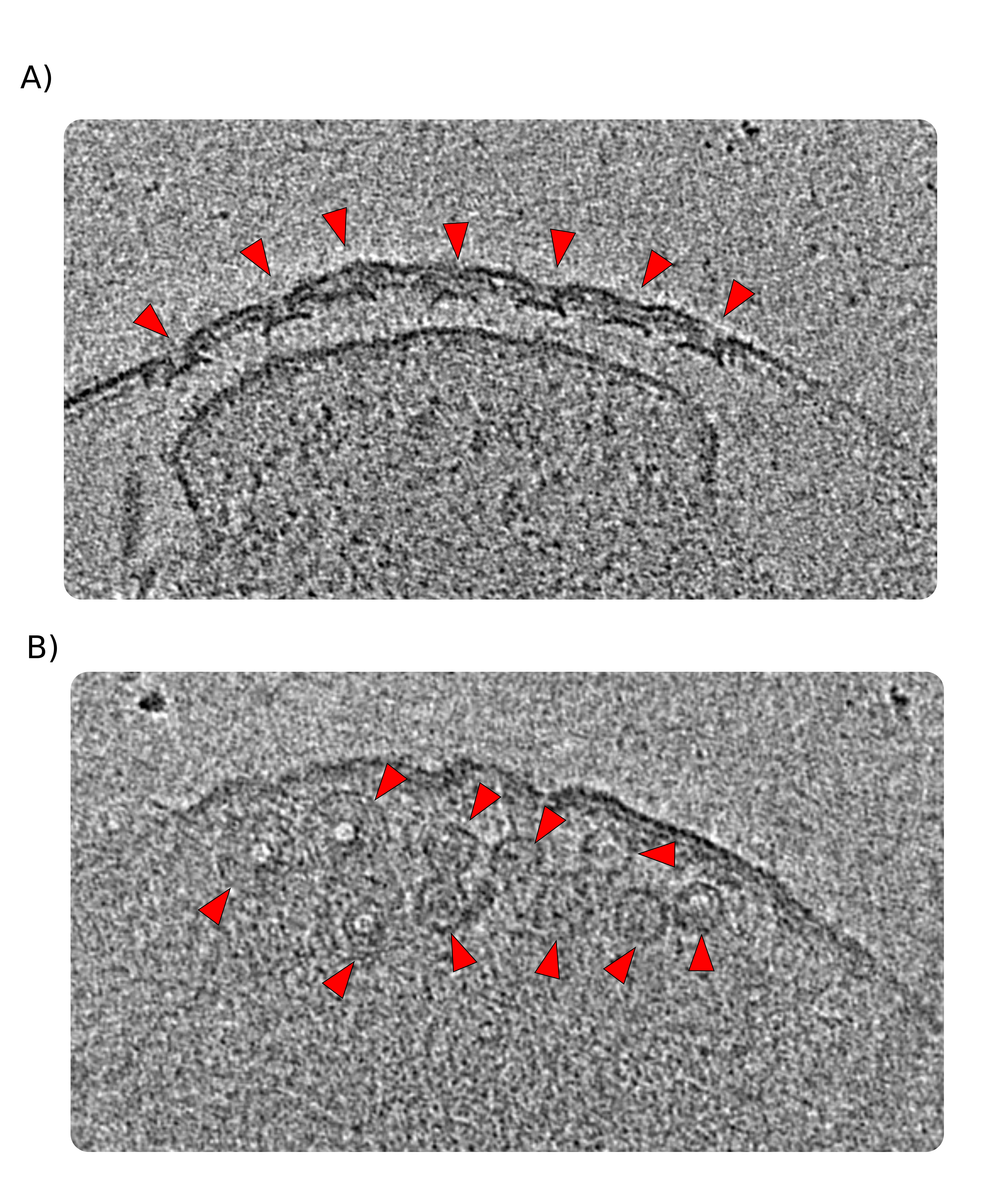

Supplement: S1 Fig — (A) Slice through a tomogram with red arrows highlighting relic structures. (B) A different slice from the same tomogram as shown in (A) showing the top view of more relic structures. (TIFF) [file pbio.3000165.s001.tiff]

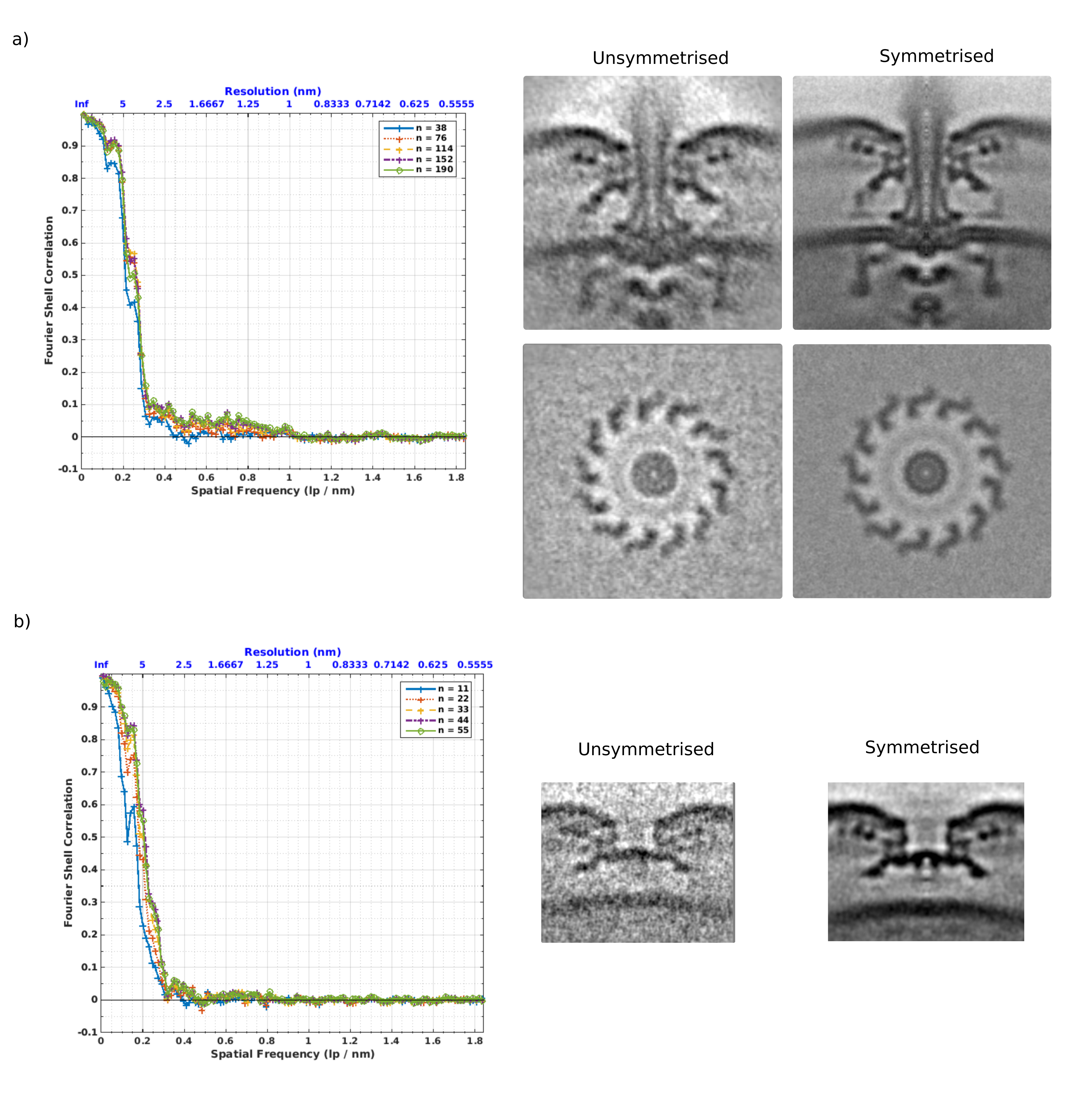

Supplement: S2 Fig — FSC curves of subtomogram averages and subtomogram averages of the P. shigelloides motor (top panel) and relic structure (bottom panel) before and after applying symmetry. Resolution at a 0.5 threshold of the unsymmetrized motor structure is approximately 3.3 nm, and the unsymmetrized relic is approximately 4.7 nm. The two bottom slices of the top panel show the 13-fold symmetry through the T-ring planes in the top central slices. FSC, Fourier Shell Correlation. (TIFF) [file pbio.3000165.s002.tiff]

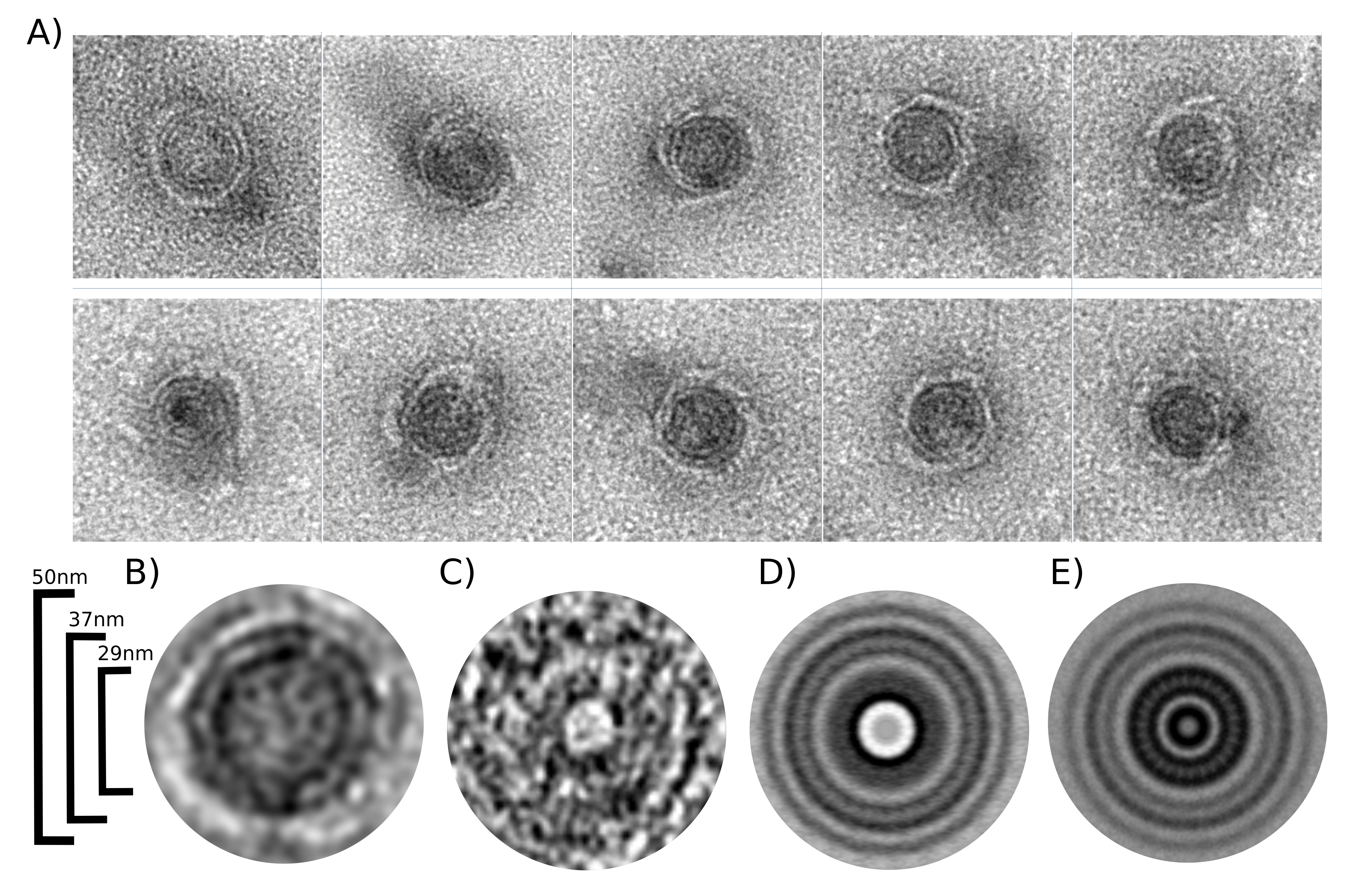

Supplement: S3 Fig — (A) Example particles extracted from negative-stain EM images of relic structures isolated using affinity purification of a S. putrefaciens MotX-His strain. (B) Example 2D class average of relic structures showing concentric rings. (C) Slice through a single tomogram of P. shigelloides showing concentric rings. (D) Slice (50 voxels thick) through the relic subtomogram average of P. shigelloides. (E) Slice (50 voxels thick) through the motor subtomogram average of P. shigelloides. EM, electron microscopy. (TIFF) [file pbio.3000165.s003.tiff]

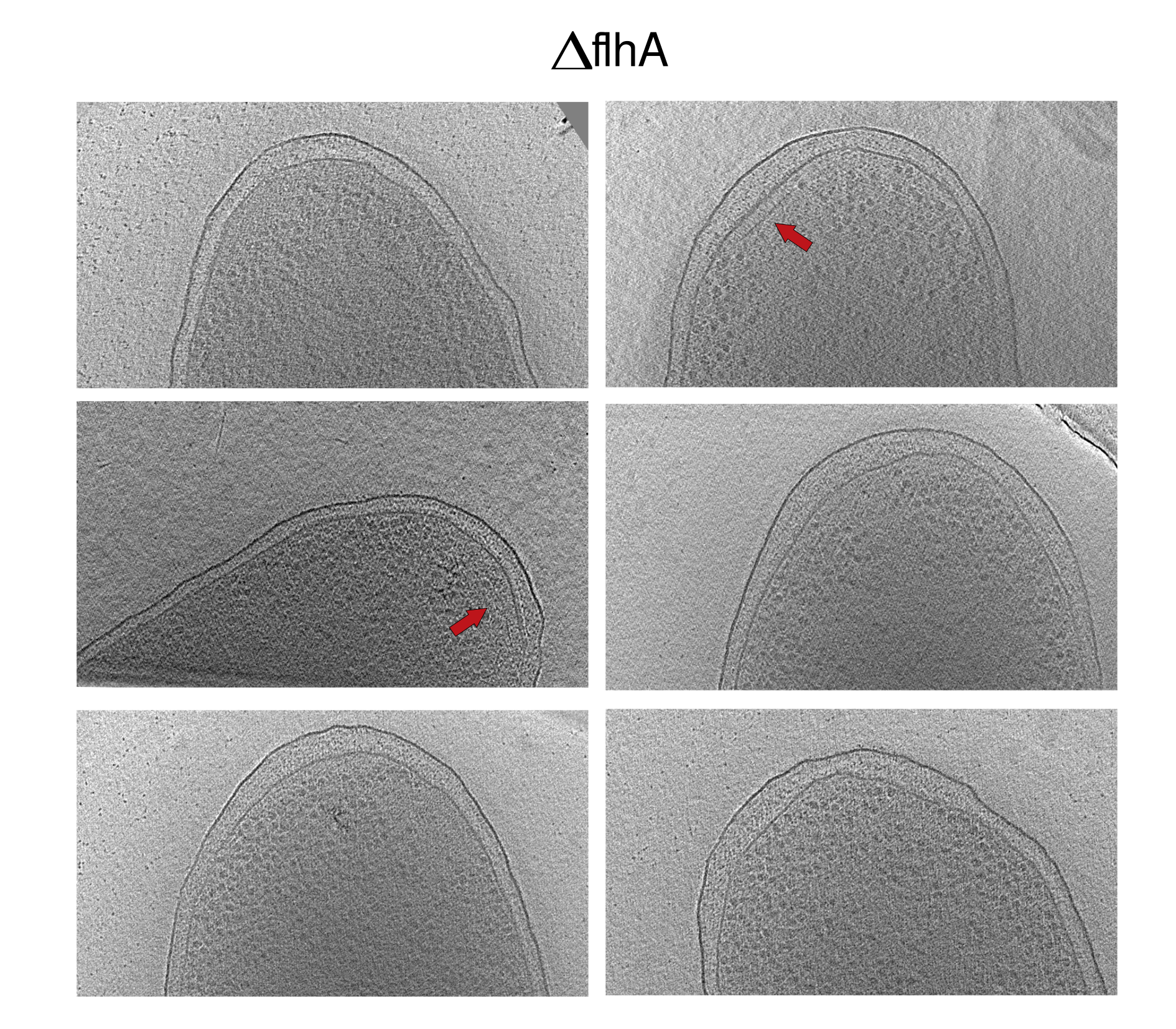

Supplement: S4 Fig — Slices through six representative tomograms of ΔflhA cells. No relics were seen at the poles of any of the 68 cells imaged. Red arrows indicate chemoreceptor arrays. (TIFF) [file pbio.3000165.s004.tiff]

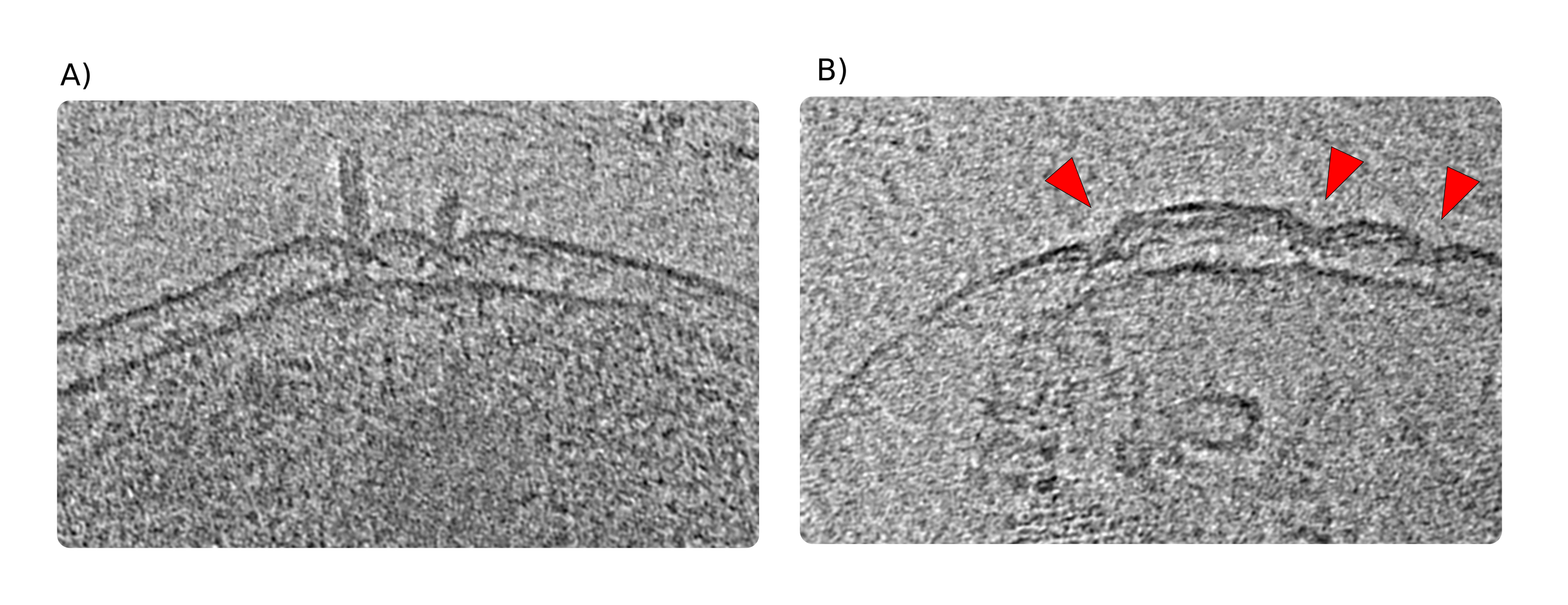

Supplement: S5 Fig — (A) Slice through a tomogram of P. shigelloides ΔfliC showing intact motors with hooks but no filament. (B) Slice through a tomogram of P. shigelloides ΔfliC showing multiple relics (red arrows). (TIFF) [file pbio.3000165.s005.tiff]

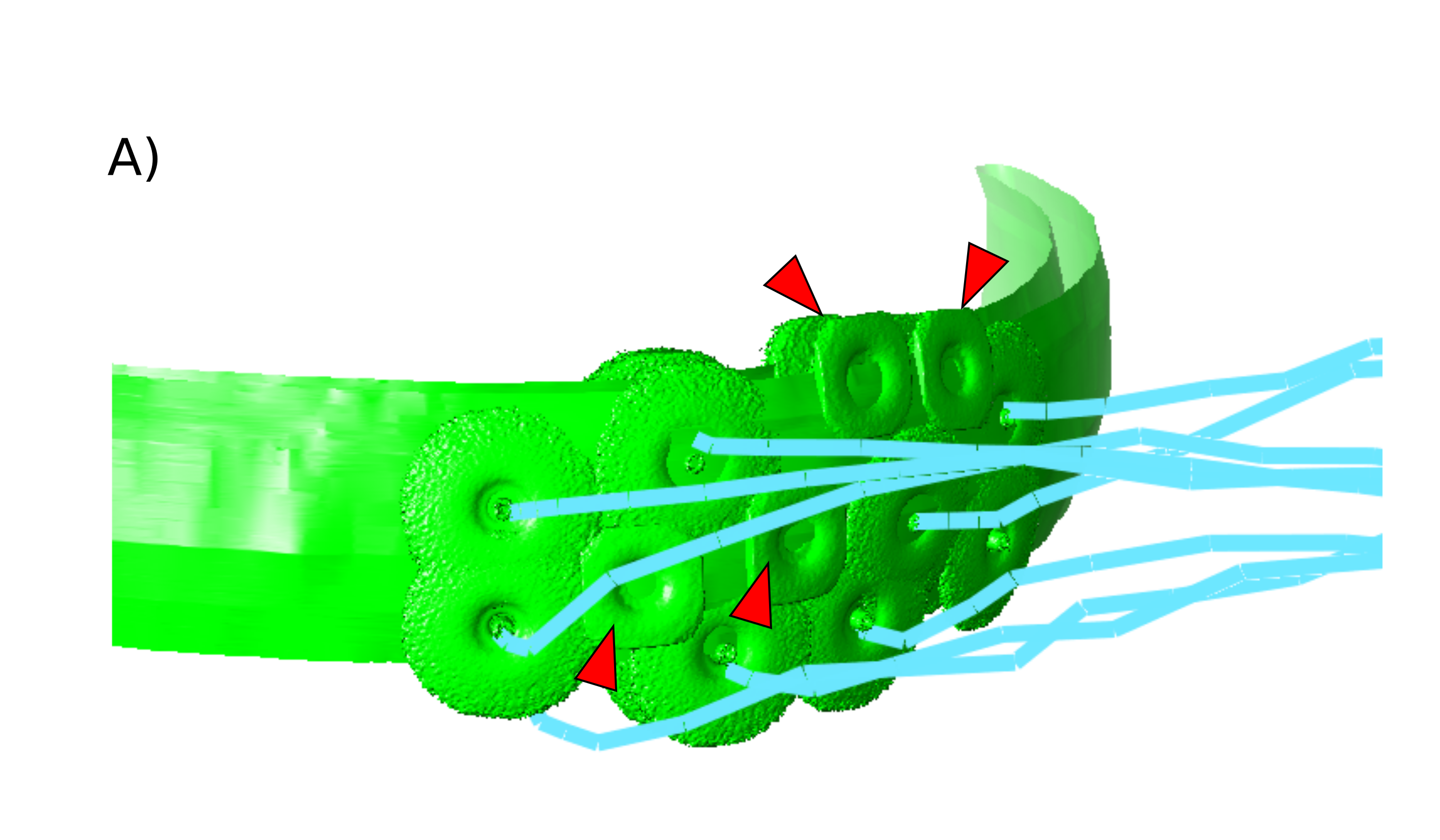

Supplement: S6 Fig — The 3D placement of relics and full flagellar motors on the pole of a representative cell. Red arrows point to relics, green flagellar filaments indicate full motors. (TIFF) [file pbio.3000165.s006.tiff]
